# Genome-wide kinase-MAM interactome screening reveals the role of CK2A1 in MAM Ca^2+^ dynamics linked to DEE66

**DOI:** 10.1073/pnas.2303402120

**Published:** 2023-07-31

**Authors:** Truong Thi My Nhung, Nguyen Phuoc Long, Tran Diem Nghi, Yeongjun Suh, Nguyen Hoang Anh, Cheol Woon Jung, Hong Minh Triet, Minkyo Jung, Youngsik Woo, Jinyeong Yoo, Sujin Noh, Soo Jeong Kim, Su Been Lee, Seongoh Park, Gary Thomas, Thomas Simmen, Jiyoung Mun, Hyun-Woo Rhee, Sung Won Kwon, Sang Ki Park

**Affiliations:** ^a^Department of Life Sciences, Pohang University of Science and Technology, Pohang 37673, Republic of Korea; ^b^Department of Pharmacology and PharmacoGenomics Research Center, Inje University College of Medicine, Busan 47392, Republic of Korea; ^c^College of Pharmacy, Seoul National University, Seoul 08826, Republic of Korea; ^d^Neural Circuit Research Group, Korea Brain Research Institute, Daegu 41062, Republic of Korea; ^e^School of Mathematics, Statistics and Data Science, Sungshin Women’s University, Seoul 02844, Republic of Korea; ^f^Department of Microbiology and Molecular Genetics, University of Pittsburgh School of Medicine, PA 15219; ^g^Department of Cell Biology, Faculty of Medicine and Dentistry, University of Alberta, Edmonton, AB T6G 2H7, Canada; ^h^Department of Chemistry, Seoul National University, Seoul 08826, Korea

**Keywords:** mitochondria-associated ER membranes, casein kinase 2, calcium, developmental and epileptic encephalopathy-66

## Abstract

The endoplasmic reticulum (ER) and mitochondria form a unique subcellular compartment called mitochondria-associated ER membranes (MAMs). Disruption of MAMs impairs Ca^2+^ homeostasis, triggering pleiotropic effects in the neuronal system. Genome-wide kinase-MAM interactome screening identifies casein kinase 2 alpha 1 (CK2A1) as a regulator of composition and Ca^2+^ transport of MAMs. CK2A1-mediated phosphorylation of PACS2 at Ser207/208/213 facilitates MAM localization of the CK2A1–PACS2–PKD2 complex, regulating PKD2-dependent mitochondrial Ca^2+^ influx. We further reveal that mutations of PACS2 (E209K and E211K) associated with developmental and epileptic encephalopathy-66 (DEE66) impair MAM integrity through the disturbance of PACS2 phosphorylation at Ser207/208/213. This, in turn, causes the reduction of mitochondrial Ca^2+^ uptake and the dramatic increase of the cytosolic Ca^2+^ level, thereby, inducing neurotransmitter release at the axon boutons of glutamatergic neurons. In conclusion, our findings suggest a molecular mechanism that MAM alterations induced by pathological PACS2 mutations modulate Ca^2+^-dependent neurotransmitter release.

## Significance

The mitochondria-associated endoplasmic reticulum membrane (MAM) is a highly dynamic structure that serves as a signaling platform for a variety of cellular activities, including Ca^2+^ homeostasis. Using a kinome-wide screening for MAM structural alterations, we identify casein kinase 2 alpha 1 (CK2A1), a catalytic subunit of casein kinase 2, as a regulator of the MAM structure and MAM Ca^2+^ crosstalk via establishing the CK2A1–PACS2–PKD2 complex. PACS2 phosphorylation by CK2A1 affects the distribution of this complex at MAMs and PKD2-dependent Ca^2+^ homeostasis. Importantly, we demonstrate that PACS2 pathogenic mutations causing the developmental and epileptic encephalopathy-66 (DEE66) disorder are associated with the disruption of PACS2 phosphorylation by CK2A1 and dysregulation of MAM Ca^2+^ dynamics, suggesting a potential therapeutic route for DEE66-associated clinical conditions.

The endoplasmic reticulum (ER) is often found in close proximity to mitochondria, forming a unique 10- to 30-nm-wide subcellular compartment called the mitochondria-associated ER membranes (MAMs) (1, 2). Increasing evidence has proposed that MAMs serve as an essential cellular signaling platform to regulate various critical cellular processes including Ca^2+^ homeostasis, lipid biosynthesis, mitochondrial biogenesis, ER stress response, inflammation, and autophagy (2). Disruption of MAMs is thought to underlie the pathogenesis of numerous diseases such as cardiomyopathy, obesity, diabetes, and neurodegenerative disorders (3–5).

Phosphorylation events have been reported to regulate the MAM structure and functions. For example, Akt/protein kinase B (6, 7), PTEN-induced kinase 1 (8, 9), Polo-like kinase 1 (10), and IRE1α (11) were individually determined to regulate MAM tethering and Ca^2+^ shuttling between the ER and mitochondria. Despite numerous studies examining the role of individual kinases in regulating MAMs, a comprehensive understanding of the regulatory pathways mediated by the human kinome is largely lacking. Using a bimolecular fluorescence complementation (BiFC)-based MAM-specific reporter generated for this study, we performed systematic, genome-wide kinase-MAM interactome screening to explore potential regulators for the structural and functional integrity of MAMs.

The screening also demonstrated that CK2A1 encoded by the *CK2A1* gene is an essential regulator of MAMs. CK2A1 is a ubiquitous serine/threonine protein kinase that plays a crucial role in mitochondrial homeostasis, mitophagy (12, 13), and apoptosis (14). Additionally, CK2A1 affects intracellular Ca^2+^ homeostasis (15) and mitochondrial fusion (16) in a Wnt/β-catenin-dependent manner. Despite evidence that suggests the involvement of CK2A1 in mitochondrial functions, the precise mechanism of how CK2A1 is associated with MAMs is unclear.

PACS2 is a multifunctional sorting protein that affects the enrichment of its cargo proteins (17, 18) on MAMs and modulates MAM functions. Specifically, PACS2 transports calnexin to MAMs to regulate Ca^2+^ oscillation at contact sites. Recently, several lines of evidence have shown that de novo *PACS2* missense variants (c.625G > A and c.631G > A) are related to a neurological disorder characterized by early onset of seizures named developmental and epileptic encephalopathy-66 (DEE66) (19–21). Interestingly, these pathological mutations of PACS2 located in CK2A1-phosphorylatable acidic clusters recognize interaction sequences for PACS family members such as PACS1 and PACS2 (22, 23). However, the relationship between CK2A1 and PACS2 at MAMs underlying the pathobiology of DEE66 requires further exploration.

Here, we identified the CK2A1–PACS2–PKD2 complex at MAMs, which plays a fundamental role in controlling MAM biology. This is achieved by phosphorylation of PACS2 at Ser 207/208/213 by CK2A1, which facilitates noncanonical PKD2-dependent Ca^2+^ transfer from the ER to mitochondria. Finally, we revealed that DEE66-related mutations of PACS2 impair the functional integrity of MAMs relevant to presynaptic neurotransmitter release in glutamatergic neurons.

## Result

### MAM-BiFC-Based Kinome Library Screening for MAM-Regulating Kinases.

We developed a MAM-reporter system using bimolecular fluorescence complementation (BiFC) for kinome library screening. Two fragments of GFP were targeted to the cytosolic face of the outer mitochondrial membrane (OMM-GFP_VC_) or ER membrane (ER-GFP_VN_) with linker repeats to ensure the typical distance between the two organelles. The correct localization and topology of the reporter on the OMM and ER membrane and the close apposition between the two organelles were confirmed (*SI Appendix*, Fig. S1*A*). Stable expression of the reporter itself did not significantly affect MAM formation (*SI Appendix*, Fig. S1 *B* and *C*) and Ca^2+^ crosstalk between ER and mitochondria (*SI Appendix*, Fig. S1*D*). As previously reported, the reporter was enhanced by ER stress induced by tunicamycin treatment (24) and reduced by high glucose (25), indicating that the reporter reflected the MAM state in response to the dynamic cellular environment (*SI Appendix*, Fig. S1*E*). These findings confirmed that the MAM-BiFC probe correctly localizes to MAMs and responds to MAM dynamics.

**Fig. 1.**
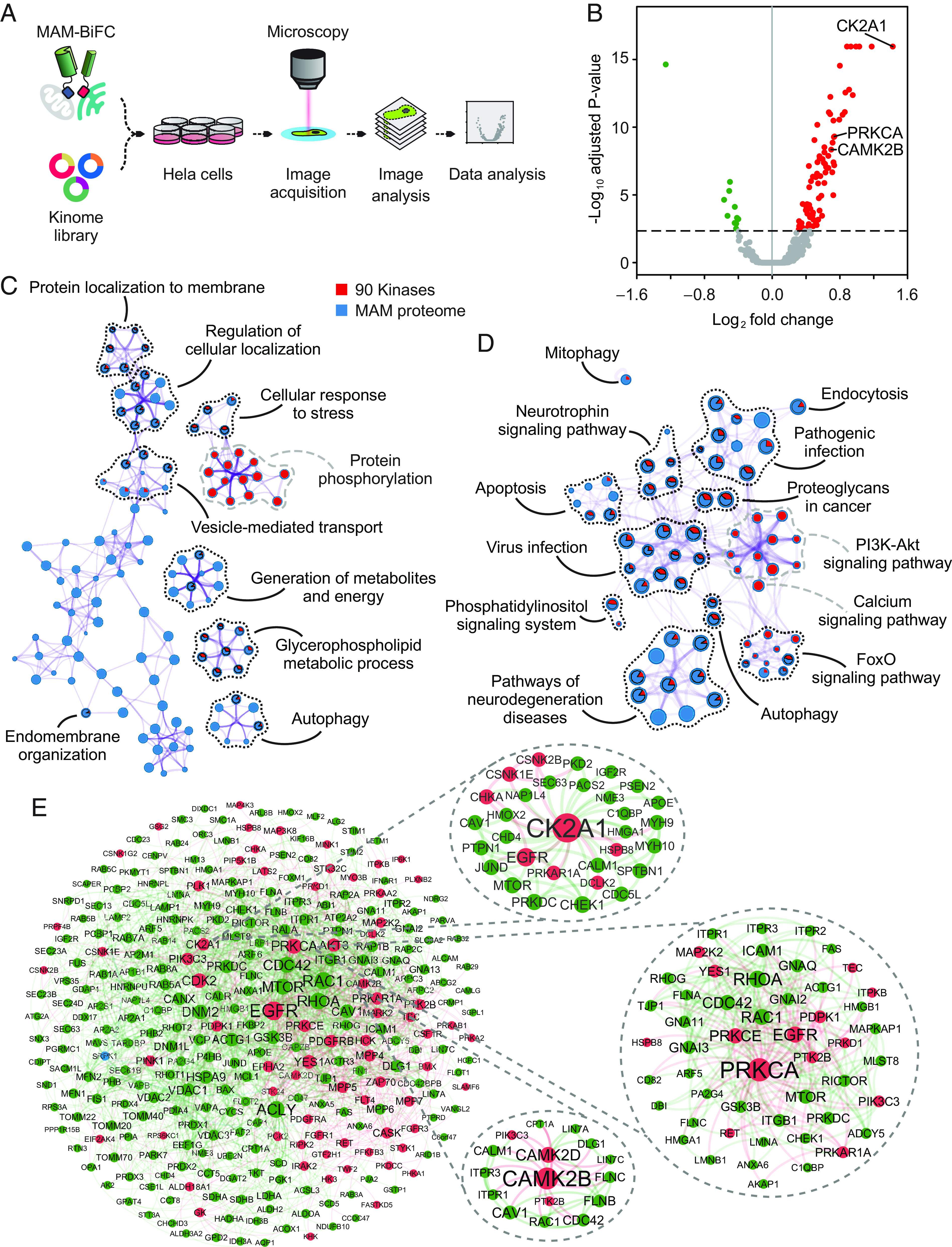
Identification of MAM regulators by human kinase-MAM interactome screening. (*A*) Schematic of screening. HeLa cells were transiently transfected with ORFs of 408 human kinases and the MAM-BiFC sensor in 12-well plates. After transfection, cells were fixed and processed for image acquisition and further analysis. (*B*) Volcano plot showing log_2_ (fold change) of MAM-BiFC signals against −log_10_ (adjusted *P*-value) in kinase-expressing cells compared with the control. The dashed line indicates adjusted *P*-value = 0.0045. Red dots indicate kinases that enhanced MAM-BiFC intensity. Green dots represent kinases that reduced the MAM-BiFC intensity. Gray dots denote kinases that did not significantly change MAM-BiFC signals. (*C* and *D*) Metascape enrichment analysis of Gene Ontology Biological Process (GO-BP) and KEGG pathways, respectively, colored by the identities of the two lists (90 kinase candidates and putative MAM-resident proteins). Nodes are displayed by pie charts whose size corresponds to the number of inputs belonging to each term. The black dotted line indicates common terms shared by the two lists. The gray dashed line indicates unique terms of the kinase candidate list. (*C*) Enrichment network visualization of GO-BP terms. (*D*) KEGG pathway terms of enriched clusters are displayed in network form. (*E*) Visualization of the protein–protein interaction network formed by the two lists using NetworkAnalyst. Subnetworks were extracted from the highlighted nodes that primarily connected to the candidate kinase of interest. Red dots denote kinases. Green dots denote MAM proteins.

To identify potential MAM regulators in the human kinome library, we coexpressed 408 kinases and kinase-related proteins with the MAM-BiFC sensor in HeLa cells (Fig. 1*A*). MAM formation was represented by the base two logarithms of the fold change (log_2_ FC) of the ratio of the MAM-BiFC intensity relative to the control (Fig. 1*B*). As a result, 90 kinase candidates, including 11 down-regulated hits and 79 up-regulated hits, were found (*SI Appendix*, Table S1).

We curated a list of MAM-resident proteins from reported MAM proteomes (*SI Appendix*, Table S2). As described in the methods section, 390 tentative MAM-resident proteins in humans and 215 human homolog proteins in mice were included. Finally, we merged all lists of MAM proteomes that comprised 534 candidates. This collection of MAM proteins and 90 MAM-regulating kinase candidates were superimposed for functional enrichment analysis. The Gene Ontology (GO) biological processes and KEGG pathways shared by the two lists are shown in Fig. 1 *C* and *D*. Notably, the majority of candidate kinases and MAM-resident proteins were involved in MAM-related biological functions such as the generation of metabolites and energy, cellular responses to stress, glycerophospholipid metabolism, and autophagy (Fig. 1*C*), indicating the reliability of the screening. They were also related to pathways of FoxO signaling, neurotrophin signaling, pathogenic infection, neurodegeneration, virus infection, and apoptosis (Fig. 1*D*). Among 90 kinases, 72 kinases had a direct interaction with MAM proteins in protein–protein interaction networks (Fig. 1*E*). In addition, the GO biological processes shared by MAM proteome and kinases that repressed MAM-BiFC signals were related to innate immune response and positive regulation of cell adhesion (*SI Appendix*, Fig. S2*A*). The GO biological processes enriched by both MAM proteome and kinases that enhanced MAM-BiFC signals mainly involved endomembrane system organization, positive regulation of protein localization, organophosphate biosynthetic process, glycerolipid metabolic process, and autophagy, among others (*SI Appendix*, Fig. S2*B*). Taken together, our genome-wide screening for MAM-regulating kinases and MAM interactome analyses provided a collection of candidate factors that likely regulate MAM formation and functions.

### Functional Validation of Selected MAM-Associated Kinases.

**Fig. 2.**
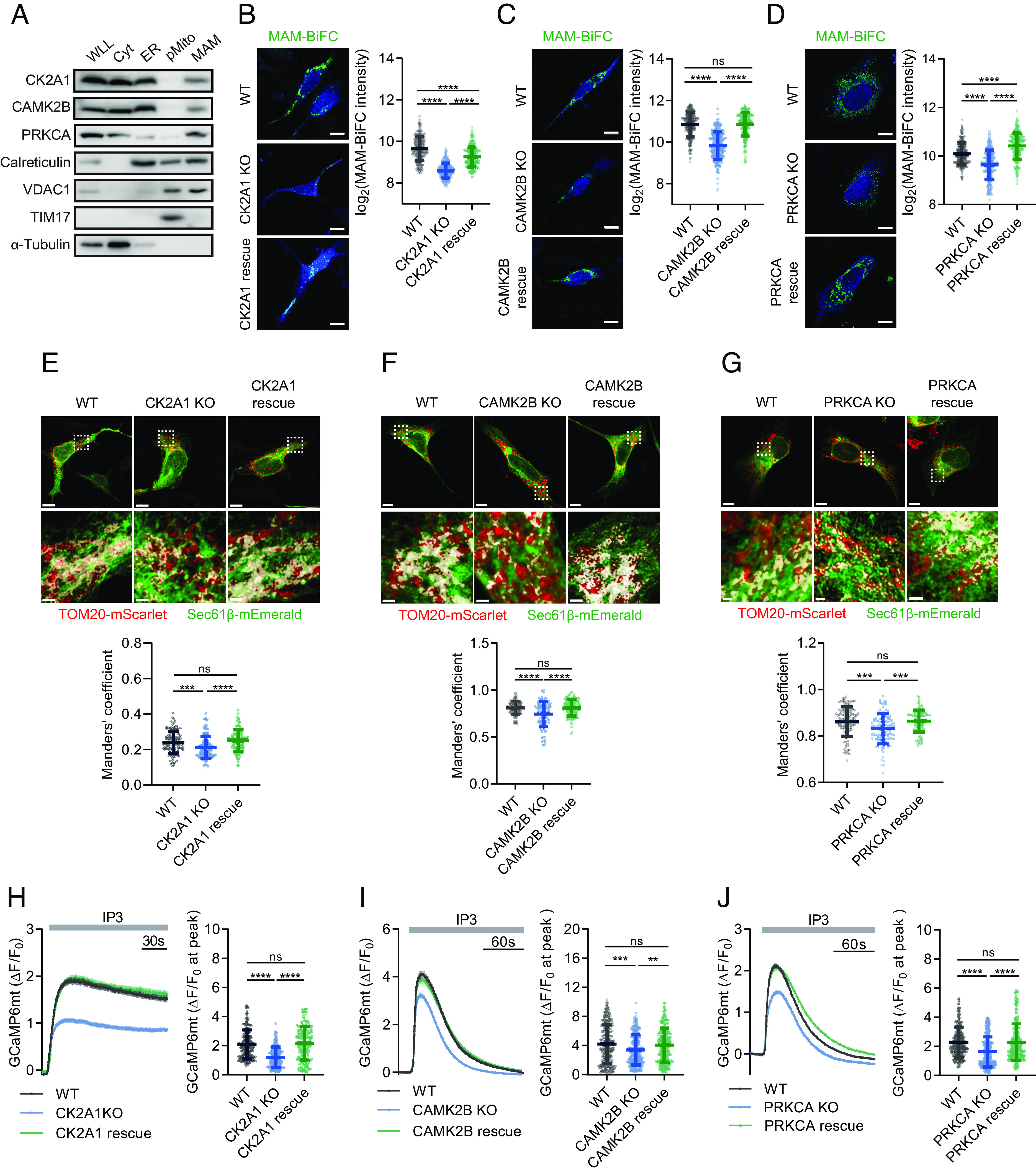
Validation of CK2A1, CAMK2B, and PRKCA kinases as MAM regulators. (*A*) Subcellular fractionation of mouse livers followed by immunoblotting analysis to detect CK2A1, CAMK2B, and PRKCA kinases at MAM fraction. WLL, whole liver lysate; Cyt, cytosol; ER, endoplasmic reticulum; pMito, pure mitochondria; MAM: mitochondria-associated membranes. (*B*) WT and HEK293 CK2A1 KO cells were transiently transfected with either the BFP vector or CK2A1-BFP and the MAM-BiFC sensor to visualize the MAM area (*Left*). Scale bar, 10 µm. *Right*, quantification plot of the MAM-BiFC intensity as the log_2_ mean intensity per cell (total cell number: WT = 198 cells; CK2A1 KO = 262 cells; CK2A1 rescue = 223 cells). (*C*) Representative images and quantification data of MAM-BiFC fluorescence in MEF WT, CAMK2B KO, and CAMK2B rescue cells. Total cells analyzed: WT = 246, CAMK2B KO = 240, CAMK2B rescue = 243. Scale bars, 10 µm. (*D*) The MAM-BiFC probe was transfected into U2OS WT, PRKCA KO, and PRKCA rescue cells, and GFP intensity was measured. Total cell number of each group: WT = 239, PRKCA KO = 237, PRKCA rescue = 231. Scale bars, 10 µm. (*E*) HEK293 WT and CK2A1 KO cells were transfected with the BFP vector, CK2A1-BFP or CK2B-BFP, in combination with Sec61β-mEmerald (ER marker) and TOM20-mScarlet (mitochondrial marker) (*Left*). Scale bars, 10 µm and 1 µm. *Right*, colocalization of the ER and mitochondria was quantified by Manders’ coefficient (total cell number: WT = 130 cells; CK2A1 KO = 146 cells; CK2A1 rescue = 125 cells). (*F*) Representative images and Manders’ coefficient value of ER and mitochondrial markers (Sec61β and TOM20, respectively) in cells are described in panel *C*. Total cell number of each group: WT = 131, CAMK2B KO= 97, CAMK2B rescue = 117. Scale bars, 7 µm, and 1 µm. (*G*) Colocalization assay of ER and mitochondrial markers (Sec61β and TOM20, respectively) in the indicated cells in panel *F*. Total cell number of each group: WT = 113, PRKCA KO = 112, PRKCA rescue = 100. Scale bars, 10 µm, and 1 µm. (*H*) Plasmids encoding mitochondrion-targeted GCaMP6s (GCaMP6mt) and either the BFP vector or CK2A1-BFP were transfected into HEK293 WT and CK2A1 KO cells. Treatment with 30 µM IP3 triggered IP3Rs-induced Ca^2+^ release from the ER, and changes in mitochondrial Ca^2+^ uptake were measured by GCaMP6mt fluorescence. *Right*, quantification plot of the maximum intensity of GCaMP6mt in each group after IP3 stimulation (total cell number: WT = 186 cells; CK2A1 KO = 176 cells; CK2A1 rescue = 187). (*I*) Mitochondrial Ca^2+^ uptake was monitored in MEF WT, CAMK2B KO, and CAMK2B rescue cells before and after IP3 treatment. Total cells analyzed: WT = 246, CAMK2B KO = 266, CAMK2B rescue = 263. (*J*) Mitochondrial Ca^2+^ entry was traced in U2OS WT, PRKCA KO, and reexpressed PRKCA cells. Total cells analyzed: WT = 262, PRKCA KO = 276, PRKCA rescue = 265. Scatter plots of panels *H*–*J* show the max peak of mitochondrial GCaMP6mt (ΔF/F_0_). Line graphs represent mean ± SEM. Data of scatter plots are presented as mean ± SD. Statistical differences were determined by one-way ANOVA and Tukey’s post hoc test for multiple comparisons. **P < 0.01, ***P < 0.001, ****P < 0.0001, ns; *P* > 0.05. All experiments were independently repeated at least three times.

To validate our screening results, we verified three high-confidence candidates (CK2A1, CAMK2B, and PRKCA) as the MAM regulators, whose expression effectively enhanced MAM formation (*SI Appendix*, Table S3). Using the CRISPR-Cas9 system, knockout (KO) cell lines were generated using HEK293, MEF, and U2OS cells in which their expression was well detected, respectively (*SI Appendix*, Fig. S3 *A*–*D*). First, we verified the existence of these three kinases in MAM fractions from mouse livers using Percoll gradient-based fractionation (Fig. 2*A*). MAM contents measured by MAM-BiFC signals (Fig. 2 *B*–*D*) and quantitative colocalization of ER and mitochondrial markers (Sec61β and TOM20, respectively) (Fig. 2 *E*–*G*) were significantly reduced in KO cells and recovered by reexpression of the three kinases. To characterize the possible regulatory role of the kinases in MAM functions, we monitored mitochondrial Ca^2+^ uptake after inducing ER-release Ca^2+^ with IP3 treatment. Mitochondrial Ca^2+^ influx was reduced remarkably in KO cells of the three kinases, and the alterations were reversed by reexpression of the corresponding kinase (Fig. 2 *H*–*J*). We found that cytosolic calcium dynamics after IP3 stimulation were unaffected by CK2A1 and CAMK2B KO (*SI Appendix*, Fig. S3 *E* and *F*). However, PRKCA KO increased cytosolic calcium signals (*SI Appendix*, Fig. S3*G*). This was consistent with the ER-release Ca^2+^ measurements after IP3 treatment in PRKCA KO and rescued cells (*SI Appendix*, Fig. S3*H*). Furthermore, among the three kinases, only CAMK2B KO lowered mitochondrial membrane potential (*SI Appendix*, Fig. S3 *I*–*K*). Our data supported the functional relevance of the kinases from screening in regulating the MAM structure and functions.

### CK2A1 Resides at MAMs and Regulates MAM Ca^2+^ Transport through the PKD2 Channel.

**Fig. 3.**
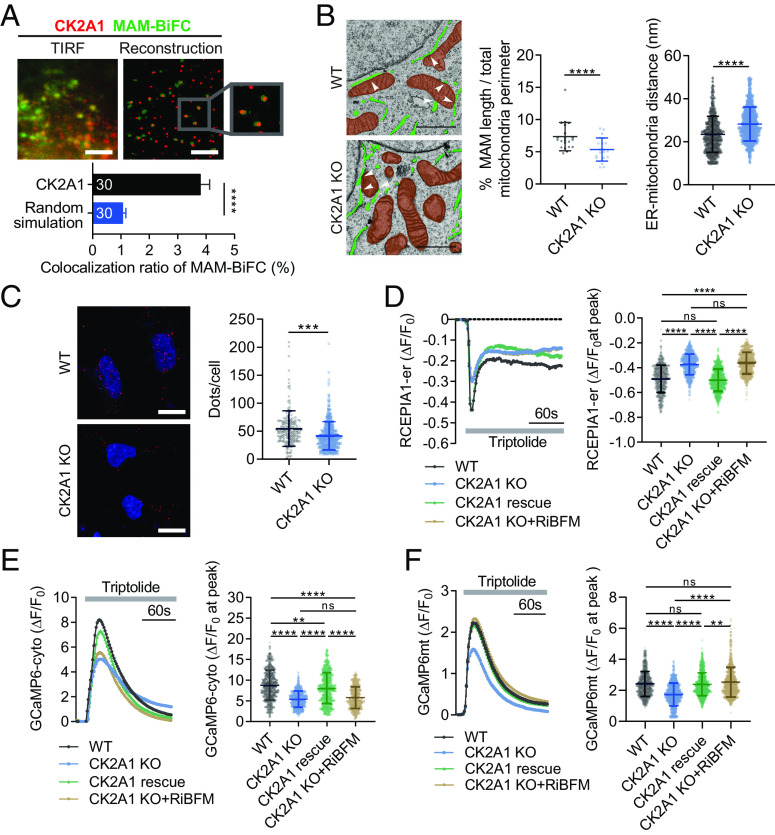
CK2A1 resides at MAMs and induces PKD2-dependent Ca^2+^ transfer between ER and mitochondria. (*A*) Representative images (TIRF panel) of a single-molecule colocalization assay of endogenous CK2A1 and the MAM-BiFC marker in HEK293 cells. Individual molecules of MAM-BiFC (green dots) and CK2A1 (red dots) were objectively detected and reconstructed using the particle detection algorithm (reconstructed panel). Lower panel, quantitative analysis of the colocalization ratio of MAM-BiFC to CK2A1. Thirty cells in each group were analyzed. Scale bar, 10 µm. (*B*) Representative TEM images of HEK293 WT and CK2A1 KO cells. The endoplasmic reticulum and mitochondria are green and brown, respectively. White arrowheads indicate MAM regions. Scatter plots show the percentage of MAM length relative to the total mitochondrial perimeter and ER-mitochondria distance, respectively (total cell number of each group = 21). Scale bars, 1 µm. Statistical significance in panels *A* and *B* was determined by a two-tailed unpaired Student’s *t*-test. ****P < 0.0001. (*C*) The PLA assay was performed using anti-VDAC1 and anti-IP3R1 antibodies in HEK293 WT and CK2A1 KO cells. Scale bars, 10 µm. The scatter plot showed the quantification of PLA-positive dots/cell. Total cell number of each group: WT = 178; CK2A1 KO = 421. (*D*–*F*) HEK293 WT and CK2A1 KO cells expressing the vector control, CK2A1, or RiBFM were used to measure calcium dynamics in mitochondria (*D*), cytosol (*E*), and ER (*F*) stimulated by triptolide using GCaMP6mt, GCaMP6cyto, and RCEPIA1-er, respectively. A total of more than 300 cells were collected and analyzed in each group. Statistical significance: **P < 0.01; ****P < 0.0001 by one-way ANOVA and Tukey’s post hoc test for multiple comparisons. Bar and line graph data represent mean ± SEM. Scatter plots show mean ± SD. Each experiment was conducted at least three times.

We focused on CK2A1, the most robust hit in the screening, by scrutinizing its detailed regulatory action for MAM functionalities. Using a single-molecule colocalization assay of MAM-BiFC signals and CK2A1 molecules with a 10 nm diffraction limit, we confirmed that endogenous CK2A1 colocalized with MAM-BiFC signals (Fig. 3*A*). Additionally, transmission electron microscopy (TEM) analysis showed the reduction of MAM in CK2A1 KO cells compared to HEK293 wild type (WT) (Fig. 3*B*). In the same line, the PLA assay using IP3R1 and VDAC1 (Fig. 3*C*), two ER and mitochondrial markers, showed a decreased proximity between ER and mitochondria in CK2A1 KO cells.

To examine the role of CK2A1 in Ca^2+^ crosstalk between the ER and mitochondria, Ca^2+^ transients of mitochondria were monitored following pharmacological activation of ER Ca^2+^ release channels. We used specific agonists, including triptolide (26), ATP (27), 4-chloro-orto-cresol (28) for polycystic kidney disease 2 (PKD2), inositol 1,4,5‐trisphosphate receptors (IP3Rs), and ryanodine receptors, respectively. Interestingly, mitochondrial Ca^2+^ uptake was dramatically reduced in CK2A1 KO cells after PKD2 or IP3R activation (*SI Appendix*, Fig. S4 *A* and *B*). We next assessed the contribution of PKD2 as the target of CK2A1 in the process of Ca^2+^ transport at MAMs by blocking IP3R-Ca^2+^ release using 2-aminoethoxydiphenyl borate (2-APB). Under this condition, 2-APB did not affect the reduction of mitochondrial Ca^2+^ uptake in CK2A1 KO cells compared with WT cells upon triptolide stimulation (*SI Appendix*, Fig. S4*C*). This result indicated that a majority of MAM Ca^2+^ transfer regulated by CK2A1 is mediated by the PKD2 channel.

To gain insights into the role of CK2A1 in Ca^2+^ crosstalk between the ER and mitochondria related to PKD2, we monitored Ca^2+^ transients of ER, cytosol, and mitochondria upon triptolide treatment in HEK293 WT and CK2A1 KO cell lines. Intriguingly, the depletion of CK2A1 diminished the ER and cytosolic Ca^2+^ response to triptolide (Fig. 3 *D* and *E*). Consistently, mitochondrial Ca^2+^ import was reduced significantly in CK2A1 KO cells (Fig. 3*F*). Additionally, when contact between the ER and mitochondria was artificially induced using the rapamycin-inducible bridge-forming module (RiBFM) in CK2A1 KO cells, the defective mitochondrial Ca^2+^ uptake was effectively restored (Fig. 3*F*), whereas no considerable changes were observed in cytosolic Ca^2+^ or PKD2-mediated ER Ca^2+^ release (Fig. 3 *D* and *E*). We observed similar results in the SY5Y cell line, a human neuroblastoma cell line (*SI Appendix*, Fig. S5 *A*–*D*). These results suggested that CK2A1 modulates mitochondrial Ca^2+^ import by regulating MAM structure and the PKD2 channel activity.

### CK2A1 Controls Mitochondrial Bioenergetics and Lipid Components Associated with the ER and Mitochondria.

**Fig. 4.**
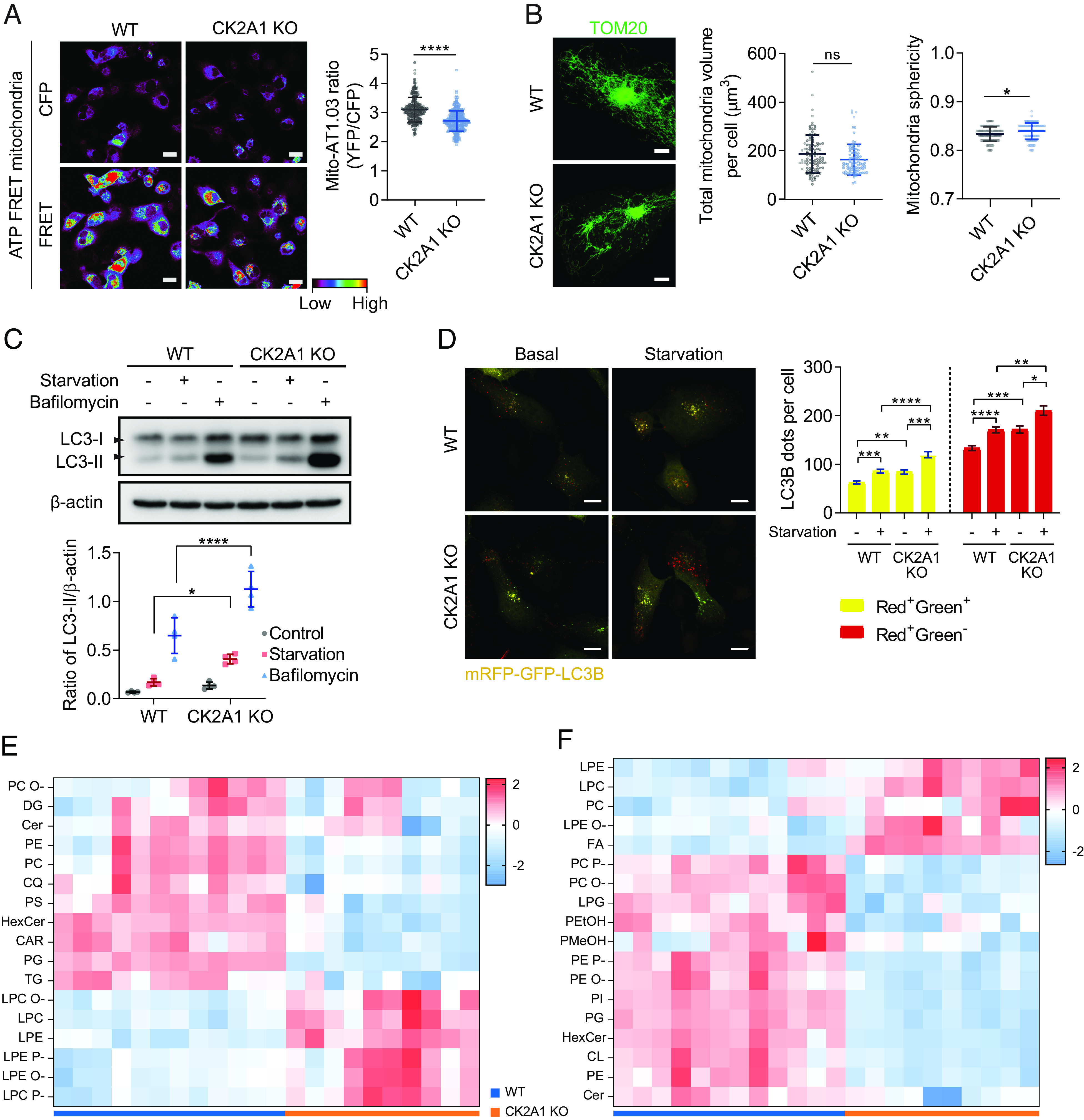
CK2A1 depletion impairs mitochondrial bioenergetics and lipid components related to the ER and mitochondria. (*A*) Mitochondrial ATP levels were evaluated as indicated by the AT01 mitochondrial FRET probe in SY5Y WT and CK2A1 KO cells. FRET imaging refers to YFP channel excitation emitted at 440 nm (*Left*). *Right*, quantification of the FRET ratio (YFP/CFP) (WT cells = 312 cells, CK2A1 KO cells = 304 cells). Scale bars, 20 µm. (*B*) The indicated cells in panel *A* were labeled for TOM20 by indirect immunofluorescence (*Left*) followed by mitochondrial morphology quantification (*Right*). The analysis included over 100 cells from each group. (*C*) SY5Y WT and CK2A1 KO cells were cultured in nutrient-rich or starvation for 2 h or bafilomycin 100 nM for 6 h and subjected to immunoblotting with antibodies against LC3 and β-actin. The scatter plot showed the quantification of LC3-II intensity relative to β-actin (n = 4). (*D*) SY5Y WT and CK2A1 KO cells expressing mRFP-GFP-LC3B were subjected to feed or starvation for 2 h and imaged using confocal microscopy. The number of RFP^+^GFP^+^ (yellow) and RFP^+^GFP^−^ (red) puncta were counted. A total of more than 150 cells of each group were collected and analyzed. The bar graph shows mean ± SEM. (*E* and *F*) Relative lipid class concentration of the purified MAM fraction from SY5Y WT and CK2A1 KO cells in positive ion mode (panel *E*) and negative ion mode (panel *F*). CAR: acylcarnitine, CL: cardiolipin, Cer: ceramide, CQ: coenzyme Q, FA: free fatty acid, DG: diacylglycerol, DG O-: ether-linked diacylglycerol, HexCer: hexosylceramide, LPC: lysophosphatidylcholine, LPC P-: ether-linked lysophosphatidylcholine (plasmalogen), LPC O-: ether-linked lysophosphatidylcholine, LPE: lysophosphatidylethanolamine, LPE P-: ether-linked lysophosphatidylethanolamine (plasmalogen), LPE O-: ether-linked lysophosphatidylethanolamine, LPG: lysophosphatidylglycerol, PC: phosphatidylcholine, PC P-: ether-linked phosphatidylcholine (plasmalogen), PC O-: ether-linked phosphatidylcholine, PEtOH: phosphatidylethanol, PE: phosphatidylethanolamine, PE P-: ether-linked phosphatidylethanolamine (plasmalogen), PE O-: ether-linked phosphatidylethanolamine, PMeOH: phosphatidylmethanol, PG: phosphatidylglycerol, PI: phosphatidylinositol, PS: phosphatidylserine, TG: triacylglycerol. Scatter plots show mean ± SD. Each experiment was repeated at least three times. Two-tailed unpaired Student’s *t*-test was used to determine statistical significance. ns, not significant; *P < 0.05; ****P < 0.0001.

We additionally investigated the impacts of CK2A1 on mitochondria-associated processes. The effects of CK2A1 depletion on ATP production were examined using a fluorescence resonance energy transfer (FRET)-based ATP sensor targeted to the mitochondrial matrix (mito-AT1.03) (29). The mitochondrial ATP level was significantly lower in CK2A1 KO cells than in WT cells (Fig. 4*A*). However, confocal microscopy revealed no significant changes in mitochondrial volume and a slight increase in mitochondrial sphericity in the CK2A1 KO condition (Fig. 4*B*). CK2A1 KO cells had increased autophagy under basal, starvation, and bafilomycin A1 (30) treatment conditions, as shown by western blotting (Fig. 4*C*). The fusion of autophagosomes to lysosomes was measured using a tandem mRFP-GFP-LC3B probe (31, 32). Under basal and starvation conditions, both immature autophagosomes (yellow puncta, RFP^+^GFP^+^) and acidified autolysosomes (red puncta, RFP^+^GFP^−^) were significantly increased in CK2A1 KO cells (Fig. 4*D*). Collectively, our data indicated that CK2A1 deficiency disrupts mitochondrial Ca^2+^ entry, causing perturbations in mitochondrial bioenergetics and induction of autophagosome formation.

We further performed high-throughput lipid profiling to examine the role of CK2A1 in lipid metabolism known to be associated with the biological functions of MAMs (33). We purified the MAM fraction and conducted the untargeted lipidomics analysis (Fig. 4 *E* and *F*). CK2A1 KO cells showed a significantly different lipid profile than WT cells. The levels of glycerophospholipids (e.g., phosphatidylcholine, phosphatidylethanolamine, phosphatidylglycerol, phosphatidylserine, and cardiolipin), ceramide, and triacylglycerol were primarily decreased in CK2A1 KO cells. On the contrary, the levels of lysoglycerophospholipids (e.g., lysophosphatidylcholine and lysophosphatidylethanolamine) and free fatty acids were significantly increased in the CK2A1 KO cells. The expression levels of individual lipid species with respect to the sum of acyl-chains and lipid subclasses are shown in *SI Appendix*, Fig. S6 and Table S4. Collectively, significant alterations of MAM lipid components are related to CK2A1 deficiency, further suggesting the essential roles of CK2A1 in regulating MAM functions.

### CK2A1 Catalytic Activity Is Crucial to Modulate Mitochondrial Ca^2+^ Influx in a PACS2–PKD2-Dependent Manner.

CK2A1 is a catalytic subunit of CK2 kinase, and therefore, we closely examined whether CK2A1 enzymatic activities are involved in MAM regulation. We reexpressed the WT or kinase-inactive form of CK2A1 (K68M) (34) in SY5Y CK2A1 KO cells. In contrast to WT CK2A1, the CK2A1 K68M mutation did not significantly enhance MAM-BiFC signals (*SI Appendix*, Fig. S7*A*) or colocalization of ER and mitochondrial markers (Sec61β and TOM20, respectively) (*SI Appendix*, Fig. S7*B*). Next, we explored CK2A1 enzymatic activities in PKD2-dependent Ca^2+^ crosstalk at MAMs. Strikingly, the inactive form of CK2A1 did not exert an apparent effect on mitochondrial Ca^2+^ transients or cytosolic and ER-released Ca^2+^ in a PKD2-dependent manner (*SI Appendix*, Fig. S7 *C* and *D*). Similarly, the inhibition of CK2A1 activity using CX-4945, a selective inhibitor of CK2A1 (35), blocked ER-release Ca^2+^ as well as cytosolic and mitochondrial calcium transient in PKD2-dependent manner (*SI Appendix*, Fig. S7 *F*–*H*). These results collectively highlighted the significant roles of enzymatically active CK2A1 in regulating the structure and Ca^2+^ transport of MAMs.

Our protein–protein interaction networks suggested potential physical interactions among CK2A1, PKD2, and PACS2 at MAMs (Fig. 1*E*). PACS2 is an essential MAM regulator that recruits its interactors residing at MAMs (17, 36). Therefore, we assessed the role of PACS2 in linking PKD2 and CK2A1 in mitochondrial Ca^2+^ import. Using shRNA constructs validated in SY5Y cells (*SI Appendix*, Fig. S8 *A*–*C*), we measured mitochondrial Ca^2+^ uptake under PKD2, PACS2, or CK2A1 deficiency. Knockdown of any of these proteins reduced mitochondrial Ca^2+^ entry to a similar extent (*SI Appendix*, Fig. S8*D*). Combined knockdown of PKD2 with CK2A1 or PACS2 or both did not cause further reduction (*SI Appendix*, Fig. S8*E*). Co-overexpression of CK2A1 and PACS2 increased mitochondrial Ca^2+^ influx, which was abolished by PKD2 knockdown (*SI Appendix*, Fig. S8*F*). This phenomenon was not due to changes in the expression of calcium-handling proteins at MAMs, such as IP3R1, VDAC1, MCU, MICU1, and MICU2 (*SI Appendix*, Fig. S8 *G*–*H*). These results suggested that CK2A1 regulates Ca^2+^ exchange at MAMs through PACS2 and PKD2.

### CK2A1 Forms a Physical Complex with PACS2 and PKD2 at MAMs.

**Fig. 5.**
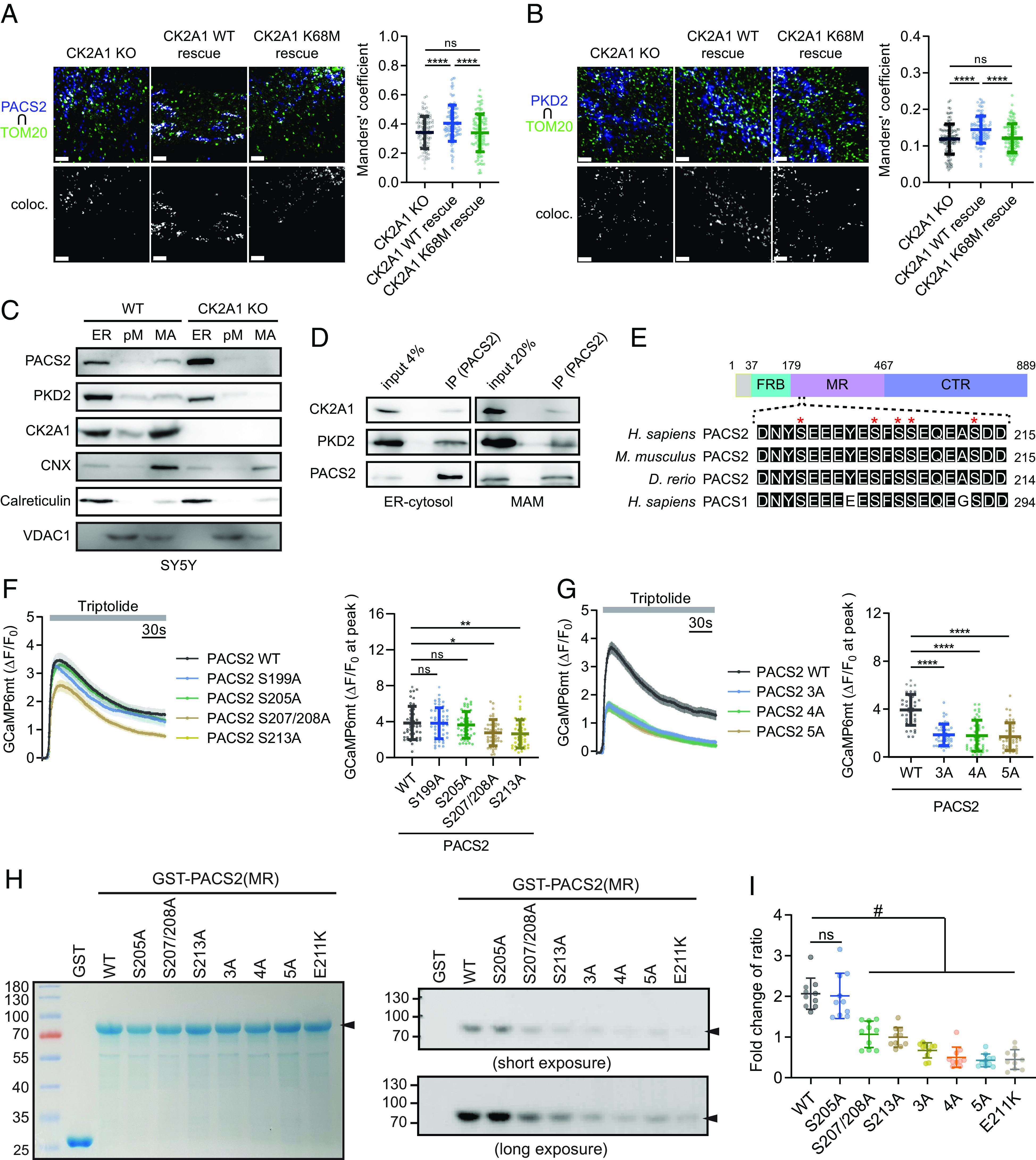
Phosphorylation of PACS2 by CK2A1 regulates the formation and Ca^2+^ homeostasis of the CK2A1–PACS2–PKD2 complex at MAMs. (*A*) Representative images of colocalization of endogenous PACS2 with TOM20 in CK2A1 KO SY5Y cells rescued by CK2A1 WT and K68M. The *Right* panel shows Manders’ coefficient of PACS2 overlapping mitochondria. Scale bars, 2 µm. Total cell number of each group: CK2A1 KO = 103, CK2A1 WT rescue = 102, CK2A1 K68M rescue = 96. (*B*) Representative images of colocalization of endogenous PKD2 with TOM20 in CK2A1 KO SY5Y cells rescued by CK2A1 WT and K68M. The *Right* panel shows Manders’ coefficient of PKD2 overlapping mitochondria. Scale bars, 2 µm. Total cell number of each group: CK2A1 KO = 113, CK2A1 WT rescue = 100, CK2A1 K68M rescue = 110. (*C*) Subcellular fractionation of SY5Y WT and CK2A1 KO cells followed by western blotting. ER markers: calnexin (CNX) and calreticulin; mitochondrial marker: VDAC1; pM: pure mitochondria; MA: MAM fraction. (*D*) Western blotting of coimmunoprecipitates using antibodies against PACS2 to detect endogenous CK2A1 and PKD2 in isolated ER-cytosol and MAM fractions. (*E*) Highly conserved acidic cluster region among species with the paralogue protein PACS1 located in the middle region of the PACS2 protein. FBR: furin (cargo)-binding region, MR: middle region; CTR: C-terminal region. Red asterisks indicate serine residues (S199-213) possibly phosphorylated by CK2A1. (*F*) Mitochondrial Ca^2+^ influx stimulated by triptolide was recorded in the somatic region of primary neurons expressing various phospho-dead mutations of PACS2 (S199A, S205A, S207/208A, and S213A). Total cells analyzed: PACS2 WT = 44, PACS2 S199A = 49, PACS2 S205A = 45, PACS2 S207/208A = 45, PACS2 S213A = 43. (*G*) Different combinations of phospho-dead mutations of PACS2 3A (S207/208/213A), 4A (S205/207/208/213A), and 5A (S199/205/207/208/213A) were transfected with GCaMP6mt into primary cortical neurons, and then, mitochondrial calcium changes were measured before and after triptolide treatment. Total cell number of each group: PACS2 WT = 39, PACS2 3A = 47, PACS2 4A = 42, PACS2 5A = 42. Scatter plots in *F* and *G* show the maximum amplitude of ΔF/F_0_ after triptolide stimulation. (*H* and *I*) In vitro kinase assay of GST-fused PACS2 middle region proteins, including WT, S205A, S207/208A, S213A, 3A, 4A, 5A, E211K, and recombinant CK2A1 protein. (*H*) The *Left* panel shows the Coomassie staining of GST-PACS2 (MR) proteins at approximately 75 kDa (black arrowhead) and GST protein at 25 kDa. Phosphorylated GST-PACS2 MR bands are shown in the *Middle* panel (short exposure time) and *Lower* panel (long exposure time). (*I*) Quantification data of the phosphorylated PACS2 (MR) band intensity normalized by protein loading. Data in line graphs demonstrate mean ± SEM. Scatter plots indicate mean ± SD. One-way ANOVA with Tukey’s post hoc tests for multiple comparisons was used to determine statistical significance. ns, not significant; *P < 0.05; **P < 0.01; ****P < 0.0001; ^#^*P* < 0.05. Each experiment was conducted at least three times.

We further examined the mechanism by which CK2A1 modulates MAM integrity and Ca^2+^ homeostasis through PACS2 and PKD2. Colocalization of either PACS2 or PKD2 with mitochondrial outer membrane marker TOM20 was considerably reduced in cells expressing the CK2A1-inactive form (Fig. 5 *A* and *B*). Comparably, MAM fractions collected from CK2A1-null cells showed reductions of both PKD2 and PACS2 (Fig. 5*C*). However, no remarkable changes were found in the expression of ER- and mitochondria-resident proteins, such as calreticulin and VDAC1, in MAM fractions. Similarly, the overall expressions of PACS2 and PKD2 were not altered in WT and SY5Y CK2A1 KO cells (*SI Appendix*, Fig. S8*G*). These results specified that the kinase activity of CK2A1 affects the MAM distribution of PACS2 and PKD2 proteins.

Next, we examined whether CK2A1 forms a complex with PACS2 and PKD2 at MAMs. Coimmunoprecipitation of CK2A1, PACS2, and PKD2 in HEK293 indicated physical interactions among these proteins (*SI Appendix*, Fig. S9 *A* and *B*). Importantly, we also found the formation of an endogenous protein complex by CK2A1, PACS2, and PKD2 in ER-cytosol and MAM fractions collected from SY5Y cells (Fig. 5*D*).

The middle region (MR) of PACS2 protein, as also seen in PACS1, consists of an acidic amino acid residue cluster (19), which is a favorable region for CK2A1 phosphorylation and highly conserved among species (Fig. 5*E*). Therefore, we hypothesized that regulation of the MAM structure and Ca^2+^ homeostasis by the catalytic activity of CK2A1 may be achieved by phosphorylation of PACS2 through this kinase. Various phospho-dead mutations of PACS2 (S199A, S205A, S207/208A, and S213A) were generated and used to clarify our hypothesis. Of note, alanine mutation at Ser207/208 or Ser213 reduced mitochondrial Ca^2+^ import via triptolide stimulation in primary cortical neurons (Fig. 5*F*). Additionally, simultaneous alanine mutation of Ser207/208 and Ser213 residues (3A) drastically decreased mitochondrial Ca^2+^ entry comparable with 4A (S205, 207/208, and 213A) and 5A mutations (S199, 205, 207/208, and 213A) (Fig. 5*G*). Next, we confirmed the phosphorylation residues of PACS2 by CK2A1 using an in vitro kinase assay. Notably, phosphorylation signals of S207/208A, S213A, 3A, 4A, and 5A were significantly reduced compared with those of the WT and S205A. Interestingly, the DEE66-related mutation, which changes an acidic, negatively charged residue to a basic, positively charged residue (E211K), located in the acidic cluster region of PACS2 MR eliminated this protein phosphorylation status by CK2A1 (Fig. 5 *H* and *I*). These findings collectively suggest a potential link between phosphorylation at Ser207/208/213 of PACS2 by CK2A1 and the pathobiology of DEE66 via regulation of the MAM structure and Ca^2+^ homeostasis.

### DEE66-Related PACS2 Mutations Alter Ca^2+^ Dynamics and Presynaptic Neurotransmitter Release.

**Fig. 6.**
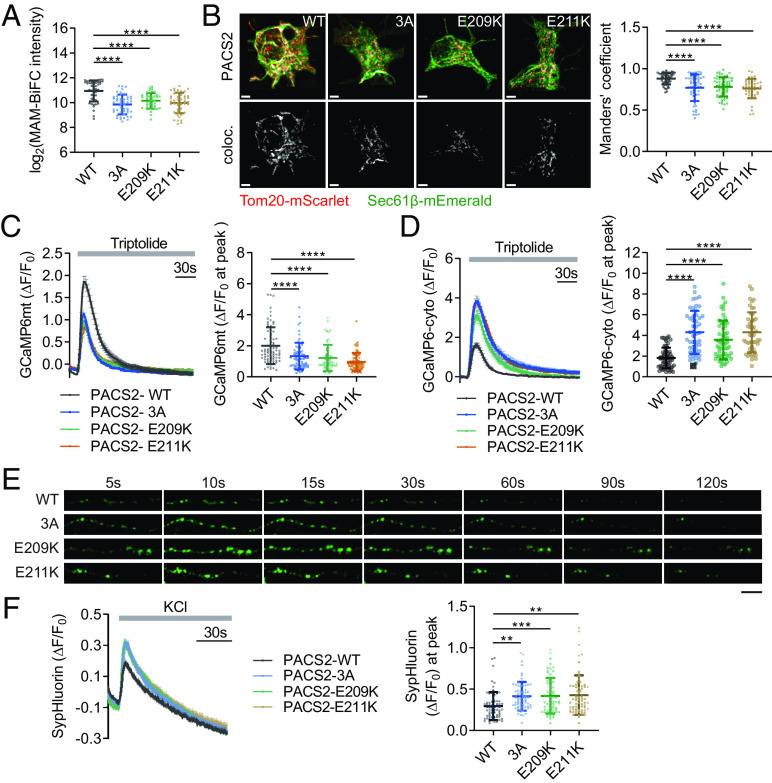
Pathological mutations of PACS2 impair MAM tethering and presynaptic PKD2-dependent Ca^2+^ homeostasis. (*A*) Quantification data of the MAM-BiFC intensity in primary neurons overexpressing PACS2 WT, 3A, E209K, and E211K. Total analyzed neurons of each group: PACS2 WT = 46, PACS2 3A = 47, PACS2 E209K = 41, PACS2 E211K = 45. (*B*) Representative images of colocalization of Sec61β and TOM20 in the soma of primary neurons expressing PACS2 WT, 3A, E209K, and E211K. Scale bar, 3 µm. *Right* panel, bar graph of Manders’ coefficient. Neuron number of each group: PACS2 WT = 51, PACS2 3A = 56, PACS2 E209K = 63, PACS2 E211K = 45. (*C* and *D*) Primary neurons expressing PACS2 WT, 3A, E209K, and E211K were monitored for changes in mitochondrial and cytosolic Ca^2+^ dynamics (*C* and *D*, respectively) stimulated by triptolide at axon boutons. Scatter plots in *C* and *D* show the maximum amplitude of ΔF/F_0_ after triptolide treatment. Total neuron number of each group: GCaMP6mt (PACS2 WT = 73, PACS2 3A = 68, PACS2 E209K = 61, PACS2 E211K = 66), GCaMP6cyto (PACS2 WT = 57, PACS2 3A = 49, PACS2 E209K = 55, PACS2 E211K = 46). (*E*) Representative images of sypHluorin fluorescence changing at axon boutons of primary glutamatergic neurons expressing PACS2 WT, 3A, E209K, or E211K before and after KCl stimulation. (*F*) Changes in sypHluorin signals (ΔF) normalized by the basal intensity (F_0_) were recorded in individual axon boutons. Scatter plot shows the maximum amplitude of ΔF/F_0_ after KCl stimulation. Total cells analyzed: PACS2 WT = 68, PACS2 3A = 64, PACS2 E209K = 67, PACS2 E211K = 67. Data in line graphs represent mean ± SEM. Scatter plots display mean ± SD. Statistical significance: *P < 0.05, **P < 0.01, ***P < 0.001, ****P < 0.0001 by one-way ANOVA with Tukey’s post hoc test for multiple comparisons. Each experiment was conducted at least three times.

Next, we determined whether phosphorylation of PACS2 at Ser207/208/213 by CK2A1 and DEE66-associated pathological mutations of PACS2 (E209K and E211K) modulate the MAM composition and PKD2-dependent mitochondrial Ca^2+^ uptake. Expression of the phospho-dead mutations of PACS2 (3A) and E209K or E211K reduced MAM formation in the quantification data of both MAM-BiFC signals and colocalization of ER and mitochondrial markers in primary cortical neurons and SY5Y cells (Fig. 6 *A* and *B* and *SI Appendix*, Fig. S10 *A*–*C*). Furthermore, in SY5Y cells, PACS2 3A, E209, and E211K mutations showed lower mitochondrial Ca^2+^ import and significantly higher cytosolic Ca^2+^ upon PKD2 activation, suggesting significant leakage of Ca^2+^ ions from MAMs under this condition (*SI Appendix*, Fig. S10 *D* and *E*). Consistently, we observed the elimination of PKD2-dependent mitochondrial Ca^2+^ uptake in axons of PACS2 3A and DEE66-related mutant-expressing neurons (Fig. 6*C*). PACS2 3A and DEE66-related mutants (E209K and E211K) significantly exaggerated the cytosolic calcium response measured by cytosolic GCaMP6s (Fig. 6*D*). Taken together, these data suggested that phosphorylation of PACS2 by CK2A1 and DEE66-related mutants alter MAM contents and diminish PKD2-dependent mitochondrial Ca^2+^ uptake, triggering an exaggerated cytosolic Ca^2+^ accumulation in cortical neurons.

**Fig. 7.**
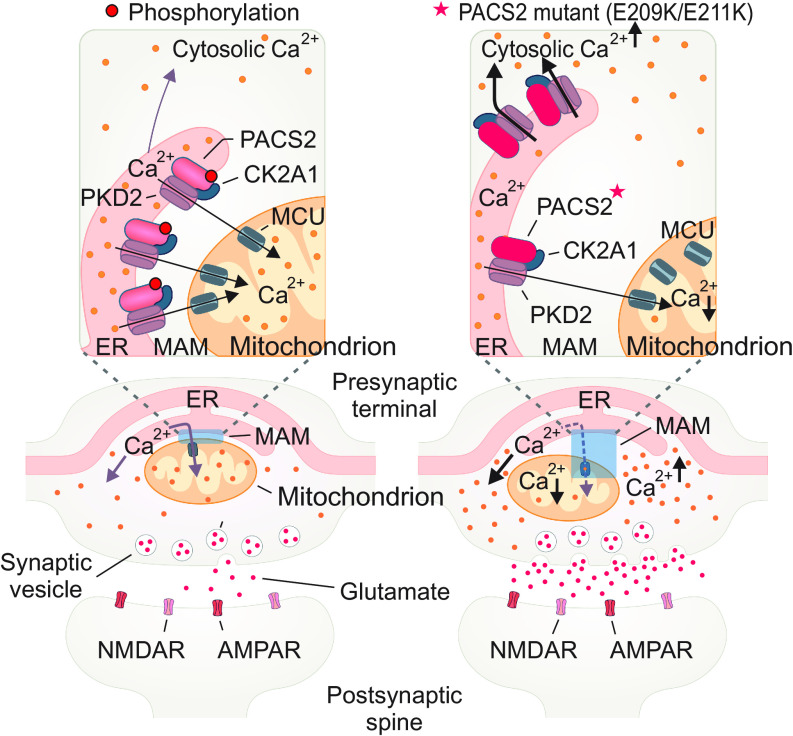
Mechanistic model for the role of the CK2A1–PACS2–PKD2 complex in the regulation of MAMs related to DEE66 pathobiology.

The local surge of cytosolic Ca^2+^ from the ER Ca^2+^ store that promoted the release of neurotransmitters in glutamatergic neurons is related to epilepsy (37). Therefore, we further examined whether the alteration of Ca^2+^ homeostasis at presynapses by the PACS2 phosphorylation state impaired the neurotransmitter release of glutamatergic neurons. To monitor neurotransmitter vesicle release of axonal boutons, we coexpressed synaptophysin-pHluorin (sypHluorin) and PACS2 phospho-dead mutation (3A) or DEE66-associated PACS2 mutations (E209K and E211K) in primary glutamatergic neurons at DIV 15-16. Remarkably, PACS2 3A as well as E209K and E211K mutations induced a significantly higher sypHluorin intensity than PACS2 WT (Fig. 6 *E* and *F*). These data revealed the effect of PACS2 pathological mutations (E209K and E211K) and the phosphorylation state of this protein at Ser207/208/213 in presynaptic neurotransmitter release of glutamatergic neurons (Fig. 7).

## Discussion

In this study, we explored *bona fide* kinases that regulate contacts between the ER and mitochondria using the MAM-BiFC sensor. We found 79 human kinases that enhanced MAM integrity. Our screening results are in agreement with many previous reports, indicating several human kinases, such as Polo-like kinase 1 (10), AKT (6), PTEN-induced kinase 1 (8), and AMPK (38), which promote MAM structural organization. Only 11 negative regulators were identified, potentially reflecting the intrinsic limitation of the BiFC system. We additionally integrated the kinase collection with various MAM proteome datasets to explore potential regulatory pathways of the MAM structure and functions. The majority of these kinase candidates have not been previously studied in MAM biology or linked to MAM components. Therefore, our screening provides a potentially important resource of regulatory signaling components that control various aspects of MAM functions. The critical role of CK2A1 in MAM functions and its link to DEE66-associated mutations of PACS2 support this notion.

We found that PACS2, a major regulatory protein of MAM tethering (39), which is also known as a PKD2 cargo protein (40), is phosphorylated at Ser207/208/213 by CK2A1 to recruit the functional CK2A1–PACS2–PKD2 complex to MAMs. Our data further indicated CK2A1 activity acts as the channel activator of PKD2, which is also supported by previous observations (41), in conjunction with the recruitment of their complex at MAMs. Indeed, PKD2 has been characterized as a channel that directly interacts with VDAC and IP3R-mediated mitochondrial Ca^2+^ entry. However, an important phenomenon of how the subcellular distribution and open-channel probability of PKD2 support MAM Ca^2+^ homeostasis regarding conventional IP3R-evoked Ca^2+^ signaling has not been addressed. Further studies will be required to clarify the binding partners of the CK2A1–PACS2–PKD2 complex to establish the calcium nanodomain at MAMs. Additionally, it will be interesting to explore the effect of CK2A1 activity in the PKD2 open-gate probability under PKD2-related clinical conditions such as autosomal dominant polycystic kidney disease (42, 43).

Here, we observed a reduction of mitochondrial Ca^2+^ uptake in CK2A1 KO cells without a detectable alteration of the mitochondrial membrane potential. This finding implies that the low Ca^2+^ import into mitochondria is not driven by changes in the mitochondrial membrane potential. Moreover, mitochondrial ATP production is boosted by the Ca^2+^ level of the mitochondrial matrix as Ca^2+^ is a cofactor of several dehydrogenase enzymes of the TCA cycle (44). Indeed, impairment of mitochondrial Ca^2+^ influx upon loss of CK2A1 leads to a shortage of mitochondrial energy generation, inducing activation of catabolic processes such as autophagy. Collectively, these findings indicate that CK2A1 fine-tunes mitochondrial physiology by mediating the structural organization and Ca^2+^ exchange at contact sites of the ER and mitochondria.

Recent studies have shown that missense mutations in the middle region of the *PACS2* gene are related to a genetic disease named developmental and epileptic encephalopathy-66 (DEE66) characterized by epilepsy, global developmental delay (with or without autism), common cerebellar dysgenesis, and facial dysmorphism (19, 20, 45). This neurodevelopmental disease is closely related to the PACS2 mutations that change acidic residues, located in the CK2A1-phosphorylated cluster, to basic residues (E209K and E211K). Remarkably, DEE66-related mutations of PACS2 not only perturb the phosphorylation state of PACS2 at Ser207/208/213 by CK2A1 but also reduce the MAM content and mitochondrial Ca^2+^ influx. The impairment of mitochondrial Ca^2+^ entry induces an overload of cytosolic Ca^2+^ in the presynaptic nerve termini. Exaggerated glutamate release at the synapse is the main pathological event of epileptic symptoms (46). In support of these findings, neurons expressing pathological mutations of PACS2 (E209K and E211K) increase neurotransmitter release at presynapses. Therefore, inhibition of CK2A1 activity or diminishing the PKD2 opening gate may be a potential therapeutic approach for DEE66-associated clinical conditions.

In conclusion, our study provides a resource for subsequent biological investigations to reveal high-confidence MAM modulators and related signaling pathways. Moreover, we demonstrate that the molecular intersection underlies the contribution of CK2A1 kinase to the regulation of the MAM structure and PKD2-evoked calcium transfer at MAMs. These mechanisms imply the potential linkage between epileptic symptoms of DEE66-related PACS2 mutations with disruption of MAM tethering and Ca^2+^ homeostasis at presynapses.

## Materials and Methods

Detailed information regarding antibodies, chemicals, and plasmid construction is provided in *SI Appendix*, *Materials and Methods*.

### Human Kinase-MAM Interactome Screening.

HeLa cells were plated and transfected with a 408 kinase ORF library alongside the MAM-BiFC sensor. Samples were fixed, mounted, and imaged using a confocal microscope. Images were processed with background subtraction and Otsu thresholding and analyzed for MAM-BiFC intensity. Data analysis involved log_2_ fold change (log_2_ FC), modified Dunnett’s test for many-to-one comparisons, and adjustment with Bonferroni correction. Putative MAM-resident proteins’ list was collected through a PubMed search, filtering by species, biospecimens, and proteomics platform. Further details of screening procedures can be found in *SI Appendix*, *Materials and Methods*.

### Protein Purification and In Vitro Kinase Assay.

The PACS2 middle region (MR) fragments were produced and purified from the *Escherichia coli* BL21 strain using glutathione–Sepharose affinity chromatography. Purified proteins were incubated with recombinant CK2 from NEB (Cat#P6010S) in a 1X NEBuffer™ containing [γ-^32^P] ATP at 37 °C for 1 h. Reactions were terminated using 5× SDS sample buffer and boiling. Samples underwent SDS-PAGE, Coomassie Blue staining, drying, and autoradiography for phosphorylated PACS2 fragment detection.

### Calcium Imaging.

Cells expressing calcium sensors (GCaMP6mt, GCaMP6-cyto, or RCEPIA1-er) and other plasmids were imaged using an inverted confocal microscope with a UPLSAPO 20×/0.75 NA objective. The medium was replaced with extracellular buffer and stimulated with various reagents (250 μM histamine, 250 μM ATP, or 200 nM triptolide). For experiments involving permeabilized cells, cells were monitored in HBSS supplemented with HEPES (pH 7.4; 2.5 mM) and treated with 6 µM ionomycin and 30 μM IP3. Images were analyzed using Cellsense software. The experiment details are available in *SI Appendix*, *Materials and Methods*.

### Proximity Ligation Assay.

Duolink^TM^ in situ proximity ligation assay (PLA) determined VDAC1 and IP3R1 interactions at MAMs. Cells were treated, blocked, and exposed to VDAC1 and IP3R1 antibodies. Following PLA probe addition, ligation and amplification occurred for 30 min at 37 °C. Images were captured using a confocal microscope with a 100× objective lens, and Imaris software quantified the number of red dots as PLA plots per cell.

Other methods are detailed in *SI Appendix*, *Materials and Methods* section. These methods include cell culture, primary neuron culture, transfection, CRISPR-Cas9 knockout cell lines, MAM fractionation, immunoprecipitation, immunoblotting, time-lapse imaging with confocal microscopy to monitor mitochondrial membrane potential, mitochondrial ATP, sypHluorin-based presynaptic vesicle release, immunocytochemistry and colocalization assay, single molecule colocalization, electron microscopy, MAM untargeted lipidomics analysis, and statistical analysis.

## Supplementary Material

Appendix 01 (PDF)

Dataset S01 (XLSX)

## Data Availability

Dataset S1 includes a list of raw lipid species from MAM lipidomics profiling in both WT and CK2A1 KO cells. All study data are included in the article and/or *SI Appendix*.
