# Supplementary material for: Genome-wide kinase-MAM interactome screening reveals the role of CK2A1 in MAM Ca2+ dynamics linked to DEE66
Source: Proc Natl Acad Sci U S A. 2023 Jul 31;120(32):e2303402120. doi: 10.1073/pnas.2303402120 (PMC10410754; doi:10.1073/pnas.2303402120)
Supplement: Supplementary file 1 — Appendix 01 (PDF) [file pnas.2303402120.sapp.pdf]

PNAS

Supporting Information for

Genome-wide kinase-MAM interactome screening reveals the role of CK2A1 in MAM Ca^{2+} dynamics linked to DEE66

Author List

Truong Thi My Nhung[1,#], Nguyen Phuoc Long[2,#], Tran Diem Nghi[1], Yeongjun Suh[1], Nguyen Hoang Anh[3], Cheol Woon Jung[3], Hong Minh Triet[1], Minkyo Jung[4], Youngsik Woo[1], Jinyeong Yoo[1], Sujin Noh[1], Soo Jeong Kim[1], Su Been Lee[1], Seongoh Park[5], Gary Thomas[6], Thomas Simmen[7], Jiyoung Mun[4], Hyun-Woo Rhee[8], Sung Won Kwon[3], Sang Ki Park[1,*]

Affiliations

[1] Department of Life Sciences, Pohang University of Science and Technology, Pohang 37673, Republic of Korea

[2] Department of Pharmacology and PharmacoGenomics Research Center, Inje University College of Medicine, Busan 47392, Republic of Korea

[3] College of Pharmacy, Seoul National University, Seoul 08826, Republic of Korea

[4] Neural Circuit Research Group, Korea Brain Research Institute, Daegu 41062, Republic of Korea

[5] School of Mathematics, Statistics and Data Science, Sungshin Women's University, Seoul 02844, Republic of Korea

[6] Department of Microbiology and Molecular Genetics, University of Pittsburgh School of Medicine, Pennsylvania 15219, USA

[7] Department of Cell Biology, Faculty of Medicine and Dentistry, University of Alberta, Edmonton, Alberta T6G 2H7, Canada

[8] Department of Chemistry, Seoul National University, Seoul 08826, Republic of Korea

* Corresponding Author Email: skpark@postech.ac.kr

[#] These authors equally contributed to this work

This PDF file includes:

Materials and methods
Figures S1 to S11
Table S1 to S4

Materials and Methods

Antibodies

Anti-CK2A1 (#sc-12738) and anti-Polycystin-2 (PKD2) (#sc-28331) mouse monoclonal antibodies were purchased from Santa Cruz Biotechnology (Dallas, TX, USA). Anti-CK2A1 (#2656S) and anti-LC3B (#2775) rabbit polyclonal antibody was purchased from Cell Signaling Technology (Danvers, MA, USA). Anti-CAMK2B (#13-9800) rabbit monoclonal antibody was obtained from Thermo Fisher Scientific (Waltham, MA, USA). Anti-PRKCA (#ab32376) rabbit monoclonal antibody was a product of Abcam (Cambridge, UK). Anti-PACS2 (#NBP1-81496) rabbit polyclonal antibody was purchased from Novus Biologicals (Littleton, CO, USA). For subcellular fractionation and subsequent immunoblotting, the following primary antibodies were used: Calnexin (Santa Cruz Biotechnology, #sc-23954), Calreticulin (Abcam, #ab2907), IP3R1 (Santa Cruz Biotechnology, #sc-28614), VDAC1 (Santa Cruz Biotechnology, #sc-8828), TOM20 (Santa Cruz Biotechnology, #sc-17764), TIM17 (Santa Cruz Biotechnology, #sc-13293), PSD95 (Enzo Life Sciences, #ADI-VAM-PS001-E). Anti-HA rabbit polyclonal (Bethyl, #A190-108A), anti-GFP rabbit polyclonal (Molecular Probes, #A-11122), anti-GFP mouse monoclonal (Santa Cruz Biotechnology, #sc-9996), anti-c-Myc mouse monoclonal (Santa Cruz Biotechnology, #sc-40), anti-α-tubulin mouse monoclonal (Proteintech Group, #66031-1-Ig), anti-GAPDH mouse monoclonal (Santa Cruz Biotechnology, #sc-32233), anti-β-actin mouse monoclonal (Sigma Aldrich, #A5441), anti-phosphoserine mouse monoclonal (Santa Cruz Biotechnology, #sc-81516), anti-MCU mouse monoclonal (Invitrogen, #MA5-24702), anti MICU1 rabbit polyclonal (Atlas Antibodies, #HPA037480), anti MICU2 rabbit polyclonal (Abcam, #ab101465) were used for IP and immunoblotting experiments. HRP-conjugated sheep anti-mouse IgG (GE Healthcare, #NA931) and donkey anti-rabbit IgG (GE Healthcare, #NA934) were used as secondary antibodies for immunoblotting. For immunostaining, Alexa Fluor 488, Alexa Fluor 647 conjugated goat anti-mouse IgG (#A11001 and #A21236), and Alexa Fluor 488 conjugated goat anti-rabbit IgG antibodies (#A11008) from Molecular Probes were used as secondary antibodies.

Chemicals

Histamine, tunicamycin, ionomycin from *Streptomyces conglobatus*, triptolide, ATP, 4-chloro-orto-cresol (4COC), oligomycin, FCCP, and bafilomycin A1 were all purchased from Sigma-Aldrich (St. Louis, MO, USA). D-myo-Inositol-1,4,5-triphosphate (potassium salt) was a product of Cayman Chem (Ann Arbor, MI, USA). For sample preparation and LC-MS data collection, toluene, methyl tert-butyl ether (MTBE), ammonium formate, ammonium acetate, and formic acid were purchased from Sigma Aldrich (St. Louis, MO, USA). Sphingosine 17:1, sphingomyelin d18:1/17:0, ceramide d18:1/17:0, triacylglycerol (TG) 17:0/17:0/17:0 and cholesterol d7 were acquired from Avanti Polar Lipids (Alabaster, AL, USA) for lipid internal standards. LC-MS grade solvents (water, 2-propanol, and methanol) were supplied by Merck (Darmstadt, Germany). Acetonitrile HPLC grade was purchased from J.T Baker (Phillipsburg, NJ, USA). CX-4945 was provided by MedChem Express (Monmouth Junction, NJ, USA). EBSS was purchased from Thermo Fisher Scientific (Waltham, MA, USA).

Plasmids

All human kinome library expression constructs were cloned into the pEZYmyc-His Gateway destination vector (Addgene plasmid #18701), which was a gift from Yu-Zhu Zhang. MAM-BiFC sensor consists of two plasmids. The N-terminal fragment of Venus domain (M1-E172), named as $GFP_{VN(1-172)}$, along with I4f linker (103 amino acids) and the ER-targeting sequence of Sac1 (from amino acids 521 to 587) was ligated to pCMV plasmid. In addition, the mitochondria targeting sequence of AKAP1 (from amino acids 1 to 30) with I2f linker (57 amino acids) and the C-terminal fragment of Venus, so-called $GFP_{VC(155-238)}$, was cloned to pCMV plasmid. CK2A1 was ligated in-frame in the plasmid pEGFP-C3 with $(GGGS)_3$ linker in between. The inactive mutation form of CK2A1 was produced by generating a point mutation that changed lysine 68 to methionine (K68M). The PACS2 with an HA tag and a pool of short-hairpin RNAs (shRNAs) against PACS2 were generously provided by Thomas Simmen. The fluorescent version of PACS2 was generated by subcloning PACS2-HA to the plasmid pmRFP1-C1. For protein

purification, the MR domain (D180-Q467) of PACS2 was subcloned to the pGEX-4T1 vector backbone. Point mutation(s) of PACS2-mRFP1 and pGEX-PACS2 MR plasmids resulted in the amino acid substitution of serine(s) for alanine(s) at residue 199, 205, 207/208, 213, 3A (S207/208/213A), 4A (S205/207/208/213A), and 5A (S199/205/207/208/213A); or substitution of a glutamic acid (E) for a lysine (K) at position 209 and 211. M-PKD2 (OF2-3) was a gift from Gregory Germino (Addgene plasmid #21370). To create PKD2 with a 6xMyc-EGFP tag, an EGFP tag was added to the N-terminus of the construct. The previously described shRNA target sites against PKD2 (1) were inserted into the pLL3.7-mRFP plasmid. As organelle markers, mEmerald-Sec61b-C1 was a gift from Jennifer Lippincott-Schwartz (Addgene plasmid #90992), and pCDNA5/tom20-pbira(79-c)-ha was constructed by Hyun-Woo Rhee (Addgene plasmid #154456). Sec61b-mScarlet and TOM20-mScarlet were prepared by changing to the desired fluorescent reporters. For calcium measurement, genetically encoded Ca^{2+} indicators GCaMP6f, jRGECO1a and RCEPIA1 added the targeting sequences for mitochondria (GCaMP6mt) or the ER (RCEPIA1-er) have been described before (2). jRGECO1a-mt plasmid was generated by subcloning MTS sequence to the pGP-CMV-NES-jRGECO1a plasmid (Addgene plasmid #61563). Cytosolic AT1.03 and mitochondria-targeted AT1.03 plasmids were gifts from Hiroyuki Noji. pcDNA3-sypHluorin 4x (S4x) was a gift from Stephen Heinemann & Yongling Zhu (Addgene plasmid #37005). mRFP-GFP-LC3B plasmid was a gift from Joo-Yeon Yoo.

Animals

Institute of Cancer Research (ICR) mice were purchased from Hyochang Science (Daegu, Republic of Korea). Embryos from E13 pregnant mice were utilized for primary cortical neuron culture, and brain lysate was prepared using 12-week-old male mice. The Pohang University of Science and Technology's Institutional Animal Care and Use Committee (IACUC) authorized all animal operations (POSTECH-2022-0085). All tests were conducted in accordance with the authorized protocols.

Cell culture, primary cortical neuron culture, and transfection

HeLa, HEK293, and SY5Y were provided by the Korean Cell Line Bank. HeLa and HEK293 cells were cultured in DMEM (HyClone, Utah, USA) with 10% (*v/v*) fetal bovine serum (FBS) (Merck) and 1% Penicillin/Streptomycin (P/S) (Thermo Fisher Scientific). SY5Y cells were cultured in MEM medium (HyClone) with 10% FBS and 1% P/S. Cells were cultured at 37 ^{o}C with 5% CO_2 and were tested for mycoplasma contamination on a regular basis. Lipofectamine 2000 (Invitrogen, #11668019) was used to transfect all cells.

Mouse embryonic cortical neurons were isolated from E13 ICR mice using 0.25% trypsin (Sigma-Aldrich) and 0.1% DNase I (Sigma-Aldrich) and maintained in the neurobasal medium (Gibco, CA, USA) supplemented with B27 (Gibco), 2mM glutamine, and 1% P/S. On the indicated day *in vitro* (DIV), cultured neurons were transfected by lipofectamine 2000 according to the manufacturer's protocol. Briefly, plasmids were mixed with lipofectamine 2000 in the neurobasal medium without any supplements, and the mixture was added to cultured neurons after 20 min of incubation. After 4 h transfection, the media were replaced with the fully supplemented neurobasal medium.

Generation of knockout cell lines using CRISPR-Cas9 system

CK2A1 KO HEK293 and SY5Y, CAMK2B KO MEF, PRKCA KO U2OS were generated using single-guide RNAs (sgRNAs) cloned into gRNA_cloning vector (Addgene plasmid #41824). Target sequences of sgRNAs included TGGGATTACGAGTCACATG and TCGTAATCCCAGTATTCTCG (for human CK2A1), TGAGGCCCTGAAGCACCCAT and CTCATGGGCCGTGATGCGCT (for mouse CAMK2B), GGGACTGGGATCGAACAACA and AGATTTCTACAGACAGTCGT (for human PRKCA). Cells were screened for EGFP to check the efficiency of each pair of sgRNAs using pCAG-EGxxFP (Addgene Plasmid #50716) containing the gRNA target sites (3). Cells were selected for 4 d after sgRNA transfection using the appropriate quantity of geneticin (Thermo Fisher Scientific, #10131035) predicted by the antibiotic kill curve. The cells were subsequently cultured at clonal density and were allowed to

develop for 10-14 days. Immunoblotting was used to examine protein expression, and probably positive clones were validated using PCR cloning and sequencing of the targeted area from genomic DNA.

Human kinase-MAM interactome screening

HeLa cells were plated on 18 mm glass coverslips coated with poly-D-lysine (50 µg/mL) and allowed to grow to 50-70% confluency. Cells were transfected by lipofectamine 2000 (Invitrogen). Each kinase from the 408 kinase ORF library was transfected with two MAM-BiFC marker fragments and incubated for 48 h. Cells were fixed in 4% PFA (Sigma-Aldrich, #158127) in PBS for 10 min at room temperature before mounting in the fluorescence mounting medium (Agilent Technologies, #S302380-2). Four hundred-eight samples were blindly marked with the serial numbers and divided into 11 batches for imaging with 11 groups of accompanied negative controls. Cells in the negative control groups were treated identically, and transfected with empty vector and MAM-BiFC markers. Each batch contained at least 37 kinases and one negative control. At least 50 cells per sample were used to gather MAM-BiFC intensity. The positive candidates inducing MAM integrity were identified by positive $\log_2$ fold change ($\log_2$ FC), and the negative candidates causing MAM loosening were indicated by negative $\log_2$ FC.

The quality of samples was strictly controlled by checking the cell morphology and MAM-BiFC signals before imaging. Cell images were obtained by the FV3000 confocal laser scanning microscope (Olympus) using a UPLSAPO 60X/1.4 NA oil objective. All the images were changed to 8-bit images, subtracted background, and performed Otsu thresholding. Otsu automatic thresholding can highlight the area where pixel values are higher than the threshold. Then, the average pixel intensity over the threshold area without background was measured. Let us define X_{ij} as a $\log_2$-transformed intensity of sample j in group i ($j = 1, \dots, n_i$, $i = 1, \dots, G$). Each group corresponds to one of kinases in the context of this paper. We denote samples in control groups by $Y_{ij}, j = 1, \dots, n_{0b}, b = 1, \dots, B$. b is used to denote different control groups from different batches. For batch b, let K_b indicate the index set of kinases obtained in batch b. The table below shows the data structure of our problem.

Batch	Control	Kinases
1	$\{Y_{11}, Y_{12}, \dots, Y_{1n_{01}}\}$	$\{X_{i1}, X_{i2}, \dots, X_{in_i}: i \in K_1\}$
2	$\{Y_{21}, Y_{22}, \dots, Y_{2n_{02}}\}$	$\{X_{i1}, X_{i2}, \dots, X_{in_i}: i \in K_2\}$
…	…	…
B	$\{Y_{B1}, Y_{B2}, \dots, Y_{Bn_{0B}}\}$	$\{X_{i1}, X_{i2}, \dots, X_{in_i}: i \in K_B\}$

Within each batch, we compared multiple kinases to a control group, which is often referred to many-to-one comparisons in statistics. Let us denote by $H_{0i}: \mu_0 = \mu_i$ the hypothesis for two-sample mean testing from control versus kinases i. Then, the multiple comparison at batch b is to test

$$H_0^{(b)} = \bigcap_{i \in K_b} H_{0i},$$

based on the observations at the b-th row. The testing can be performed by a modified Dunnett's test proposed by Herberich and colleagues (4).

Many-to-one comparisons and its hypothesis can be expressed as the general linear hypothesis under the one-way ANOVA model. Also, an asymptotic theory of mean estimators under the model is available even when group sizes are unequal, and heteroscedasticity exists. As a consequence, the virtue of this statistical procedure is being free from strong assumptions required for the classical Dunnett's test, i.e., Gaussianity, equal group sizes, and homogeneity across groups, which are usually non-realistic in the settings of biological experiments. The test provided k_b(the number of kinases in batch b) P-values that are adjusted to control the family-wise error rate for $H_0^{(b)}$, not for the whole null hypothesis $H_0 = \bigcap_{1 \leq b \leq B} H_0^{(b)}$. Therefore, we needed another adjustment of those P-values to satisfy the overall significance level of testing. In this additional adjustment, we simply relied on Bonferroni correction. In other words, we say the intensity of at least one of kinases in batch b is significantly different from that of a control group (significance level at α) if $\mathrm{P}_b < \alpha/B$, where P_b is the individual P-value

computed from batch b. α was set to be 0.05 in our study. The whole process was implemented using R *multcomp* package.

MAM proteome collection

A systemic search in PubMed was conducted to collect the studies that identified MAM proteome using the terms: "(mitochondria or mitochondrial) and (ER or endoplasmic reticulum) and (contact or contacts or mitochondria-associated membrane or mitochondria-associated membranes or MAM or MAMs or mitochondria-ER contacts or MERCs) and ("proteomic profiling" or "proteomic analysis" or "proteomic characterization" or proteome or proteomics or proteomic or labeling)". The literature search was finished on 1 June 2020. Then, the references to relevant articles were reviewed to identify relevant studies for further assessment. A study was qualified when it had the criteria below: (1) species (*Homo sapiens, Mus musculus*), (2) biospecimens (cell lines, tissues), (3) proteomics platform (mass spectrometry), and (4) well-validated MAM proteins confirmed by multiple experiments and reported by individual research articles. Data extraction was conducted according to the workflow as shown in Fig S11. (1) Studies were separated by species (*Homo sapiens*, *Mus musculus*). (2) The majority of studies were conducted in *Homo sapiens* using mammalian cell lines (HEK293T, fibroblasts, PH5CH8, and Huh7). Another group included all studies using *Mus musculus* tissues (brain, retina, testis, liver). (3) Methods to enrich MAM proteins included Percoll gradient biochemical fractionation and proximity labeling (APEX, split APEX, split-BioID, contact-ID).

Subcellular fractionation of MAMs

Subcellular fractionation was carried out to isolate ER, MAM, or mitochondria as described previously (5, 6). Briefly, harvested cells were rinsed with solution A (0.32 M sucrose, 1 mM $NaHCO_3$, 1 mM $MgCl_2$, 0.5 mM $CaCl_2$, protease inhibitor cocktail). Cells were homogenized and centrifuged at 1400 *g* for 5 min at 4 °C. A part of the supernatant was preserved as a whole lysate fraction, while the remainder was centrifuged for 10 min at 13,800 *g*, 4 °C. The crude MAM pellet (MAM+Mito) was resuspended in solution B (0.32 M sucrose, 1 mM $NaHCO_3$, protease inhibitor cocktail). The supernatant, on the other hand, was placed into a discontinuous sucrose gradient and centrifuged for 70 min at 152,000 *g*, 4 °C. The white band was collected using a syringe and centrifuged for 45 min at 126,000 *g*, 4 °C, with the pellet collected as the ER fraction. The preceding crude MAM pellet, resuspended in solution B, was put on a discontinuous sucrose gradient containing 1 mM $NaHCO_3$ and centrifuged for 2 h at 82,500 *g*, 4 °C. The third white band was collected with a syringe as the microsomal fraction, and the resultant pellet was reconstituted in an isolation medium (250 mM mannitol, 5 mM HEPES pH 7.4, 0.5 mM EGTA, 0.1% BSA) and put on top of Percoll gradient (225 mM mannitol, 25 mM HEPES pH 7.4, 1 mM EGTA, 30% Percoll). Upper and lower bands were collected as MAM and mitochondrial fraction, respectively.

MAM fractionation using mouse livers was conducted by differential ultracentrifugation using the Percoll gradient according to a previous report (6). All the fractions including whole liver lysate, cytosol, ER, pure mitochondria, and MAMs were collected, lysed in 1X modified RIPA lysis buffer, measured protein concentration using BCA assay (Thermo Fisher Scientific, # 23228), and subjected to immunoblotting experiments.

Western blot analysis

Cells were collected and lysed in 1X modified RIPA lysis buffer (50 mM Tris pH 7.5, 150 mM NaCl, 1% NP-40, 5 mM EDTA, 1% Triton X-100, 0.5% sodium deoxycholate) supplemented with 2 mM NaPPi, 10 mM NaF, 2 mM Na_3VO_4, 1 mM DTT, protease inhibitor cocktail (Thermo Fisher Scientific, #A32963). For immunoblotting analysis, proteins were denatured by mixing lysates with 5X SDS sample buffer (1 M Tris-Cl pH 6.8, glycerol 50%, SDS 10%, bromophenol blue 0.5%, DTT 0.5 M) and incubating at 70°C for 15 min. SDS-PAGE with 7-13% polyacrylamide gel was used to separate proteins, which were

then transferred to a PVDF membrane (Millipore, Billerica, MA, USA). Membranes were blocked with 5% skim milk or 5% bovine serum albumin (BSA) in Tris-buffered saline (20 mM Tris pH 8.0 and 137.5 mM NaCl) with 0.25% Tween20 (TBST) for 1 h before being incubated with primary antibodies overnight at 4 °C and then HRP-conjugated secondary antibodies for 1 h at room temperature. ECL solutions recognized protein signals (BioRad, Hercules, CA, USA).

Immunoprecipitation assays

Cells were lysed in 1X Triton X lysis buffer (50 mM Tris-HCl pH 7.4, 150 mM NaCl, 1% Triton X-100, 1 mM EDTA) supplemented with phosphatase inhibitor cocktail (Thermo Fisher Scientific, #A32957), and protease inhibitor cocktail (Thermo Fisher Scientific, #A32963). Lysates were incubated with 1 µg of antibody at 4 °C for 16 h with rotation. Protein A agarose beads (Roche, #5015979001) were rinsed three times with 1X Triton X lysis buffer and combined with immunoprecipitated lysates and rotated at 4 °C for at least 4 h. The beads were collected via centrifugation and washed three times with 1X Triton X lysis solution before being combined with the SDS sample buffer for carryout out the immunoblotting analysis.

Protein purification and *in vitro* kinase assay

Recombinant GST-fused PACS2 middle region (MR) fragments were expressed and purified from the BL21 strain of *Escherichia coli* using glutathione-sepharose affinity chromatography (GE Healthcare) as previously described (7). Each purified protein was incubated in the presence of purified recombinant CK2 purchased from NEB (Cat#P6010S). Reactions were carried out in a 1X NEBuffer™ for protein kinases (50 mM Tris-HCl, 10 mM $MgCl_2$, 0.1 mM EDTA, 2 mM DTT, 0.01% Brij 35, pH 7.5) containing [γ-^{32}P] ATP (10 µCi) at 37 ^{o}C for 1 h and then terminated by adding 5X SDS sample buffer and boiling for 10 min. Samples were subjected to SDS-PAGE, stained by Coomassie Brilliant Blue, and dried, and then phosphorylated PACS2 fragments were detected by autoradiography.

Immunofluorescence and colocalization assay

Cells expressing Sec61β-mEmerald and TOM20-mScarlet or stained with Alexa 488 and Alexa 647-conjugated antibodies (Molecular Probes, dilution factor; 1:200) were analyzed using an FV3000 confocal laser scanning microscope (Olympus) with a UPLSAPO 60X/1.4 NA oil objective, 488-nm laser line and 543-nm / 647-nm laser line. Each channel's photomultiplier gain was adjusted to reduce background noise and saturated pixels. The 3D deconvolution was completed with Imaris software CUDA Deconvolution extension (Bitplain, Version 9.1.2). ImarisColoc was utilized to automate the selection of the thresholds, measure the number of co-localized voxels, and ROI that was co-localized, and calculate Manders' coefficient.

Single-molecule colocalization

Glass coverslips (18 mm) were prepared as previously mentioned (8). Then, coverslips were coated with poly-D-lysine (50 µg/mL) dissolved in PBS for 1 h before seeding SY5Y expressing MAM-BiFC. After 12 h, the cells were fixed with 4% paraformaldehyde in PBS for 10 min at room temperature, washed three times with PBS, permeabilized with 0.05% Triton X for 3 min, and then blocked with 4% BSA for 1 h. In order to identify endogenous CK2A1, cells were treated with primary antibodies at room temperature for 3 h, followed by indirect immunofluorescence labeling with Alexa Fluor 647-conjugated secondary antibodies. On a handmade objective-type total internal reflection fluorescence (TIRF) microscope constructed on an inverted microscope (IX-81, Olympus) with an XYZ automated stage, fluorescence imaging was performed (MS-2000, Applied Scientific Instrumentation). A 561-nm laser (YLK 6150T, Lasos) was aligned with an oil-immersion TIRF objective lens (APON 100X/1.49 NA, Olympus). An electron-multiplying charge-coupled device (EM-CCD) camera (iXon Ultra 897, Andor Technology) equipped with an adapter was used to capture images (TuCam, Andor Technology). To improve the magnification, a 1.6X amplifier, and a 1.43X tube lens were used. All instrument operations and data acquisitions were managed by MetaMorph (Molecular Devices) and MATLAB plug-ins that were developed specifically for this study (MathWorks). The eternity buffer was prepared as previously

described (8) for inducing the photoblinking of Alexa Fluor 647, and its signal was excited using the 642-nm laser (VFL-P-1000-642, MPB Communications) at 30 W/cm^2 with a frame rate of 0.05 Hz in the far-red channel (654-870 nm). GFP signal was excited using a 488-nm laser (543-BS-A03, Melles Griot) at 10 W/cm^2 with a 0.05 Hz frame rate in the green channel (500-549 nm). Multiple particle detection method was performed based on U-track as previously described (9). To eliminate the false positives (MAM-BiFC with the random colocalized coordinate), we compared the distribution of the colocalization frequency of MAM-BiFC to their randomizing coordinates. The randomization function and quantitative analysis were written in Matlab (The MathWorks).

Electron microscopy

Cells were grown in 35 mm glass-bottomed culture dishes to 50%-60% confluency. Cells were fixed with 2 ml of the fixative solution containing 2% paraformaldehyde and 2.5% of glutaraldehyde diluted in sodium cacodylate buffer (pH 7.2) at 4 °C. After being washed, then post-fixed in 2% osmium tetroxide (OsO4) containing 1.5% potassium ferrocyanide for 1 h at 4 °C. The fixed cells were dehydrated using an ethanol series (50%, 60%, 70%, 80%, 90%, and 100%) for 20 min at each concentration and infiltrated with an embedding medium. Samples were cut horizontally to the plane of the block by 60 nm (Leica Microtome GmbH, Vienna, Austria) and were mounted on copper slot grids with a specimen support film and double-stained with lead nitrate and 2% uranyl acetate. The samples were then inspected using a transmission electron microscope Tecnai G2 (Thermo Fisher Scientific, Waltham, MA, USA). The 16 mosaic images per cell were analyzed using the lasso selection tool of Microscopy Image Browser (MIB) (10), ER, mitochondria, and MAM areas were selected and converted to masks corresponding to object identification. MAM was quantified using two different approaches as calculated the percentage of MAM length per total mitochondrial perimeter (11) and the distance between ER and mitochondrial membrane.

Proximity ligation assay

DuolinkTM *in situ* proximity ligation assay (PLA) was used to measure the interaction between VDAC1 and IP3R1 at the ER–mitochondria contacts, following a previously described protocol (12, 13). Cells were fixed and blocked, then incubated overnight at 4 °C with primary antibodies against rabbit anti-VDAC1 (Proteintech, #55259-1-AP) and mouse anti-IP3R1 (Santa Cruz, #sc-271197). The PLA probes against the specific primary antibodies host were applied for 1 h at 37 °C. Then, ligase was added for 30 minutes at 37 °C, and amplification was completed. Lastly, cells were mounted after Hoechst staining. Images were obtained using an FV3000 confocal laser scanning microscope (Olympus) with a UPLSAPO 100X/1.35 NA oil objective. The PLA signals were detected by Texas red-labeled probes and indicated ER–mitochondrial contact points. The number of red dots as PLA plots per cell was quantified using Imaris software.

Calcium imaging

Cells were transfected with GCaMP6mt, GCaMP6-cyto, or RCEPIA1-er, along with respective expression constructs or shRNA plasmids for 72 h. Following transfection, the medium was replaced with extracellular buffer containing HEPES (pH 7.4; 25 mM), $CaCl_2$ (2 mM), $MgSO_4$ (1 mM), $NaHCO_3$ (4 mM), and D-glucose (30 mM). Depending on the experiment's purpose, cells were treated with indicated reagents (250 μM histamine, 250 μM ATP, or 200 nM triptolide) and subsequently imaged at intervals of 2 s using FV3000 confocal laser scanning microscope (Olympus) with a UPLSAPO 20X/0.75 NA objective. For experiments involving permeabilized cells, cells were treated with 6 μM ionomycin in HBSS supplemented with HEPES (pH 7.4; 2.5 mM) for 3 min before imaging. After 10 s of imaging, cells were then applied 30 μM IP3 and imaged for another 145 s. Primary neurons transfected with GCaMP6mt or GCaMP6-cyto along with PACS2-related constructs were imaged in an extracellular buffer. Neurons were treated with 400 nM triptolide after baseline recordings. Images were analyzed using the Cellsense software (Olympus). The whole cell area or neuronal soma was selected by rectangle ROI by Cellsense, and average intensities were measured with background correction. After intensities were corrected for background subtraction, ΔF values were calculated from ($F-F_o$). F values displayed the intensities at peak while F_o values were defined by averaging 5 frames before stimulation and used for normalization.

TMRM imaging

Cells were incubated with 20 nM tetramethylrhodamine methyl ester (TMRM) (Invitrogen, # T668) in HEPES-buffered HBSS for 30 min at 37 °C. Measurements were obtained by using FV3000 confocal microscope with a UPLSAPO 20X/0.75 NA objective while keeping a bath of 5 nM TMRM in the imaging solution. TMRM was excited using the 543-nm laser line and fluorescence intensity was recorded. Basal mitochondrial membrane potential as well as dynamic changes upon application of oligomycin and FCCP were measured at a single focal plane. The intensity profile was collected by Cellsense software. TMRM was used in the redistribution mode, meaning that a reduction in TMRM fluorescence represents mitochondrial depolarization.

Mitochondrial ATP determination

Mito-AT1.03 probe-transfected cells were stimulated at 405 nm and CFP and YFP emissions were collected concurrently at 475 nm and 525 nm for FRET-based ATP determination in mitochondria. The YFP/CFP fluorescence intensity ratio was used to determine the mitochondrial ATP level in each cell.

SypHluorin imaging

To monitor presynaptic neurotransmitter vesicle release from axonal boutons, synaptophysin-pHluorin (sypHluorin) was co-expressed with PACS2 phospho-dead mutation (3A) or human DEE66-associated PACS2 mutations (E209K, E211K) in primary glutamatergic neurons at DIV 15-16. KCl 50 mM was used to chemically induce synaptic activity. SypHluorin signals were analyzed by the Cellsense software (Olympus). After background subtraction, ΔF values were deduced from (F-F_o) where F displayed the intensities at peak following KCl stimulation while F_o indicated the baseline values.

Untargeted lipidomics analysis

SY5Y WT and CK2A1 KO cells were cultured as mentioned above, seeded into a 15 cm tissue culture dish at a density of 7 × 10^6 cells per plate, and harvested 72 h after seeding. Cells were collected and subjected to differential ultracentrifugation using the Percoll gradient to obtain the MAM fraction as described previously (6).

Approximately 20 mg of the fraction pellet was used for extracting lipids. In particular, lipids were extracted using the MTBE method (14, 15). Firstly, 250 µL MeOH (-80 °C) was added to the sample and then homogenized using Precellys 24 at 5500 rpm x 15 s x 2, resting on dry ice for 2 min between each cycle. The sample was transferred to a new tube and 850 µL MTBE (-20 °C) was subsequently added and vortex for 60 s. The sample was shaken at 1500 rpm, 4 °C for 1 h. Next, 210 µL H2O was added, vortex for 60 s before additionally incubating on a shaker for 15 min at 4 °C. Next, samples were centrifuged at 16000 *rcf* x 10 min at 4 oC. The upper layer (400 µL x 2) was transferred to another tube and dried under nitrogen purge at room temperature until completely dry. Lipid extract was stored at -80 °C until analysis. For untargeted lipidomics analysis, the sample was suspended in 80 µL of MeOH:toluene (9:1 v/v), and a post-extraction pooled QC sample was created by mixing 20 µL of each sample.

The data acquisition was performed using Thermo Q Exactive Plus instrument following our established protocol (14) developed based on Fiehn's lab method (15). Sample was injected to an Acquity UPLC CSH C18 column (100 × 2.1 mm; 1.7 µm) connected with an Acquity UPLC CSH C18 VanGuard pre-column (5 × 2.1 mm; 1.7 µm) (Waters, Milford, MA, USA). For injection volume, 1 µL was injected in positive ion mode and 4 µL in negative ion mode. Column was maintained at 65 °C and run with 0.6 mL/min flow rate. The mobile phase employed in this study included mobile phase (A) acetonitrile:water (60:40, v/v) with ammonium formate (10 mM) and formic acid (0.1%) and (B) 2-propanol:acetonitrile (90:10, v/v) with ammonium formate (10 mM) and formic acid (0.1%) in positive mode. The negative mobile phases consisted of (A) acetonitrile:water (60:40, v/v) with ammonium acetate (10 mM) and (B) 2-propanol:acetonitrile (90:10, v/v) with ammonium acetate (10 mM). We applied the following gradient for lipidomics analysis: 0 min 85% A; 0-2 min 70% A; 2-2.5 min 52% A; 2.5-11 min 18% A; 11-11.5 min 1% A; 11.5-12 min 1% A; 12-12.1 min 85% A; 12.1-16 min 85% A. Regarding mass

spectrometer parameters, the instrument was run with the following parameters: MS1 mass range: 120-1200, resolution: 70000 FWHM (*m/z* 200), AGC target: 1e6, MAXIMUM IT: 100 ms. For dd-MS/MS, machine was run with following parameters: resolution: 17500 FWHM (*m/z* 200), AGC target: 1e5, MAXIMUM IT: 50 ms; Loop count, 4; TopN, 4; Isolation Window, 1.0 *m/z*; (N)CE/stepped (N)CE, 20 in positive mode and 20, 30, 40 in negative mode; Spectrum data type, profile.

MS-DIAL was applied to data processing and lipid annotation (16). Lipid annotation was conducted using our in-house authentic library and *in silico* MS/MS library. Lipid matches with experimental *m/z*, retention time tolerance of 0.5 min and *in silico* spectral was identified as the highest identification level. Lipid nomenclature was presented following the recent LIPID MAPS classification update (17).

Annotated lipid was used to further data analysis and visualization. Data were first filtered to remain only the peaks with a relative standard deviation (RSD) in QC less than 20% and then processed with median normalization and log transformation before statistical analysis. Principle component analysis (PCA) and heatmap were constructed using MetaboAnalyst 5.0 (18). Lipid class analysis was conducted on Lipidsig in two separated ion modes with the log transformation setting (19).

Statistical analysis

Quantitative data were presented as Mean and SD unless otherwise stated. The sample size varied depending on the properties of the experiments. Statistical analyses were conducted by either R 4.2 statistics or GraphPad Prism 9.3.1. A two-tailed independent-samples t-test was employed to determine whether the means of two independent groups were significantly different. One-way or two-way analysis of variance (ANOVA) and Tukey's post hoc test were applied to compare the means of more than two separate groups. As the level of statistical significance, a criterion of 0.05 was adopted unless otherwise stated. For lipidomic analysis, significant lipids were considered with an adjusted P-value (i.e., False-Discovery Rate) < 0.05.

Figure S1 - S11

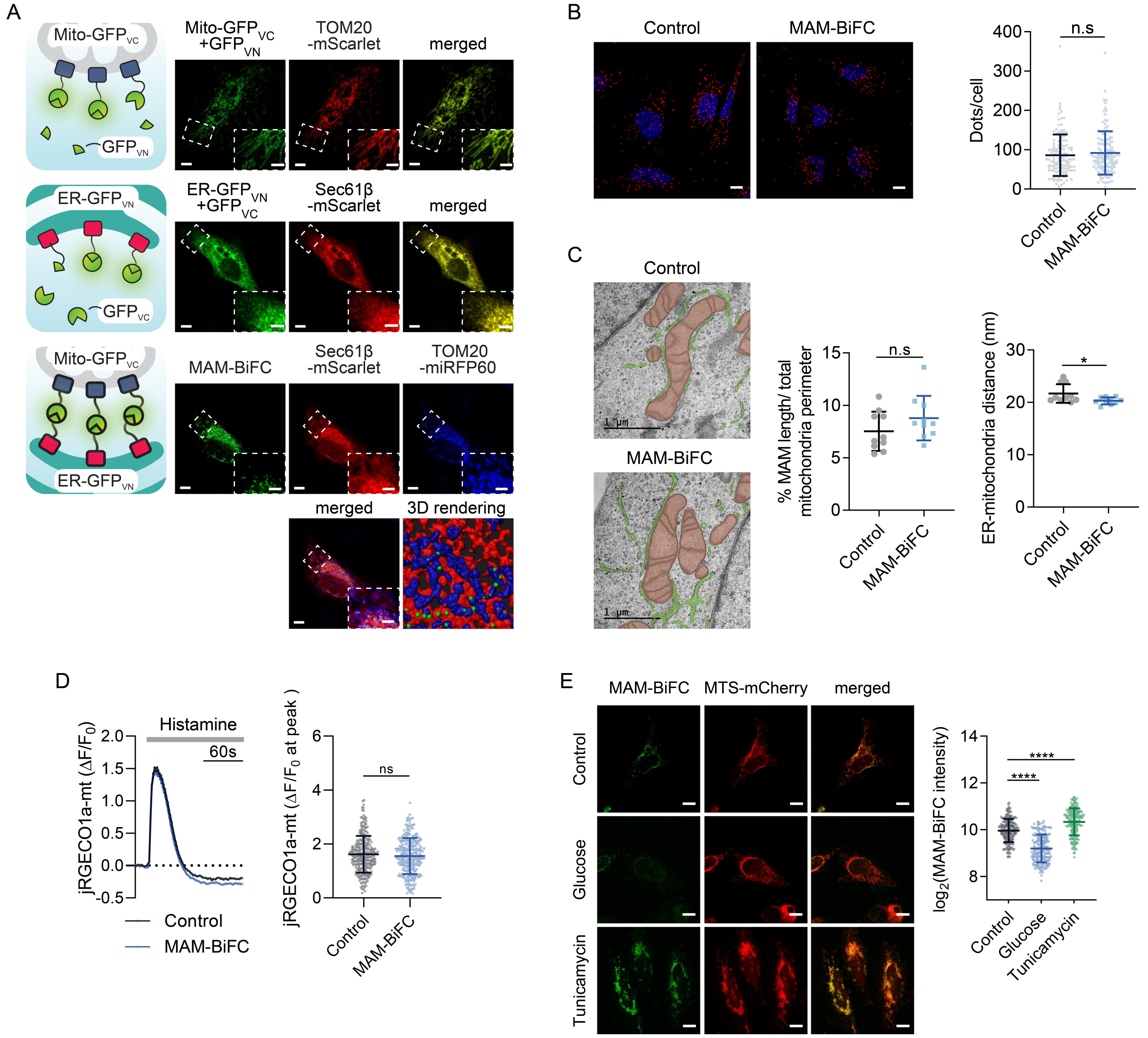

Fig S1. Characterization of the MAM-BiFC marker. (A) Illustration and representative images of the general approach to validate the correct targeting of the MAM-BiFC probe. The correct mitochondrial pattern and ER network were verified by co-expression of cytosolic GFP_{VN} and Mito-GFP_{VC} (upper panel), of cytosolic GFP_{VC} and the ER-GFP_{VN} plasmid (middle panel). Co-transfection of two fragments of the MAM-BiFC probe (Mito-GFP_{VC} and ER-GFP_{VN}) induced GFP dots located at close contacts of the ER marker (Sec61β-mScarlet) and mitochondrial outer membrane marker (TOM20-miRFP670) (lower panel). High-magnification images were processed, deconvoluted, and rendered in 3D using Imaris analysis software. Scale bars, 10 μm and 2 μm for higher magnification. (B) Representative images of PLA assay using anti-VDAC1 and anti-IP3R1 antibodies in control and MAM-BiFC expressing HeLa cells. Scale bars, 10 μm. (Right panel) Quantification of PLA-positive dots/cell in A. Total cell number of each group: control = 130; MAM-BiFC = 128. (C) The indicated cells in panel B were imaged using TEM to visualize MAMs. The ER and mitochondria were colored green and brown, respectively. (Right panels) Two quantification methods to quantify the changes of MAMs in EM images. Scale bars, 1 μm. Total of 10 cells from each group were analyzed. (D) Mitochondrial Ca^{2+} uptake in control and MAM-BiFC expressing HeLa cells were recorded before and after histamine treatment. Scatter plot shows the peak amplitude of $\Delta F/F_o$. Total cell number of each group: control = 263, MAM-BiFC = 266. Statistical significance in panel

B-D was determined by two-tailed unpaired Student's t-test. Ns, not significant; *P<0.05. (E) Changes in MAM-BiFC signals were determined in HeLa cells expressing the MAM-BiFC marker and MTS-mCherry cultured in normal, high glucose, or tunicamycin conditions. Scatter plot shows quantification data of the MAM-BiFC intensity. Scale bars, 10 μm. Statistical significance in panel E: ****P<0.0001 by one-way ANOVA and Tukey's post hoc test for multiple comparisons. Total cells analyzed: control = 191, glucose = 188, tunicamycin = 192. Line graph presents mean ± SEM. Scatter plots indicate mean ± SD. Each experiment was conducted at least three times.

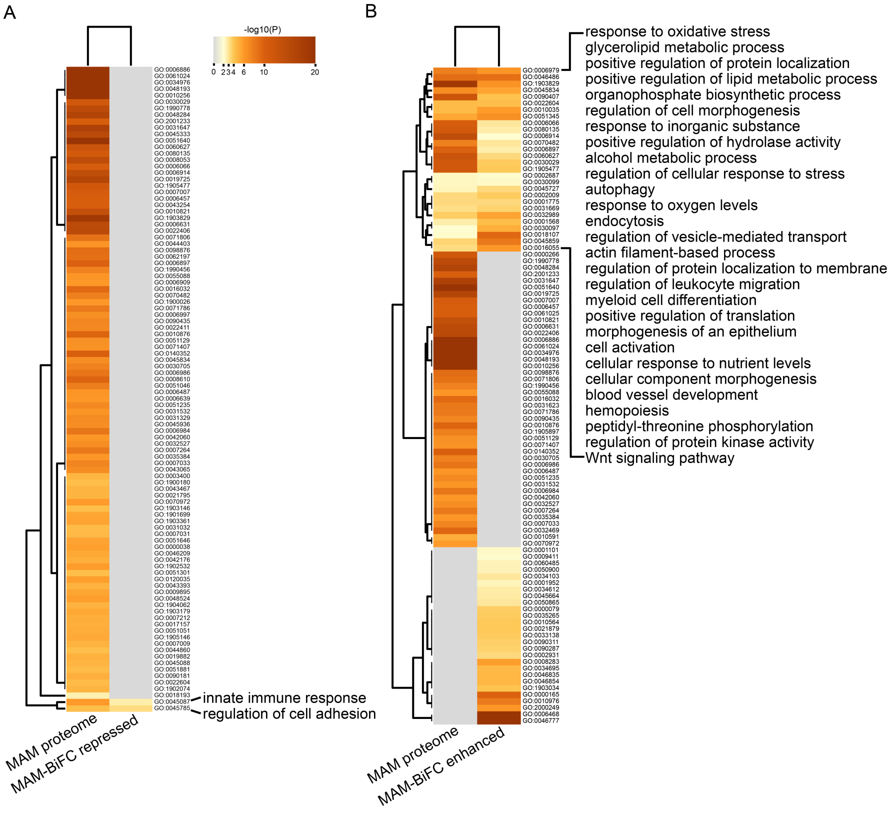

Fig S2. Heatmaps showing Gene Ontology Biological Process enrichment analysis of putative MAM-resident proteins and kinases that repressed (A) or enhanced (B) MAM-BiFC signals.

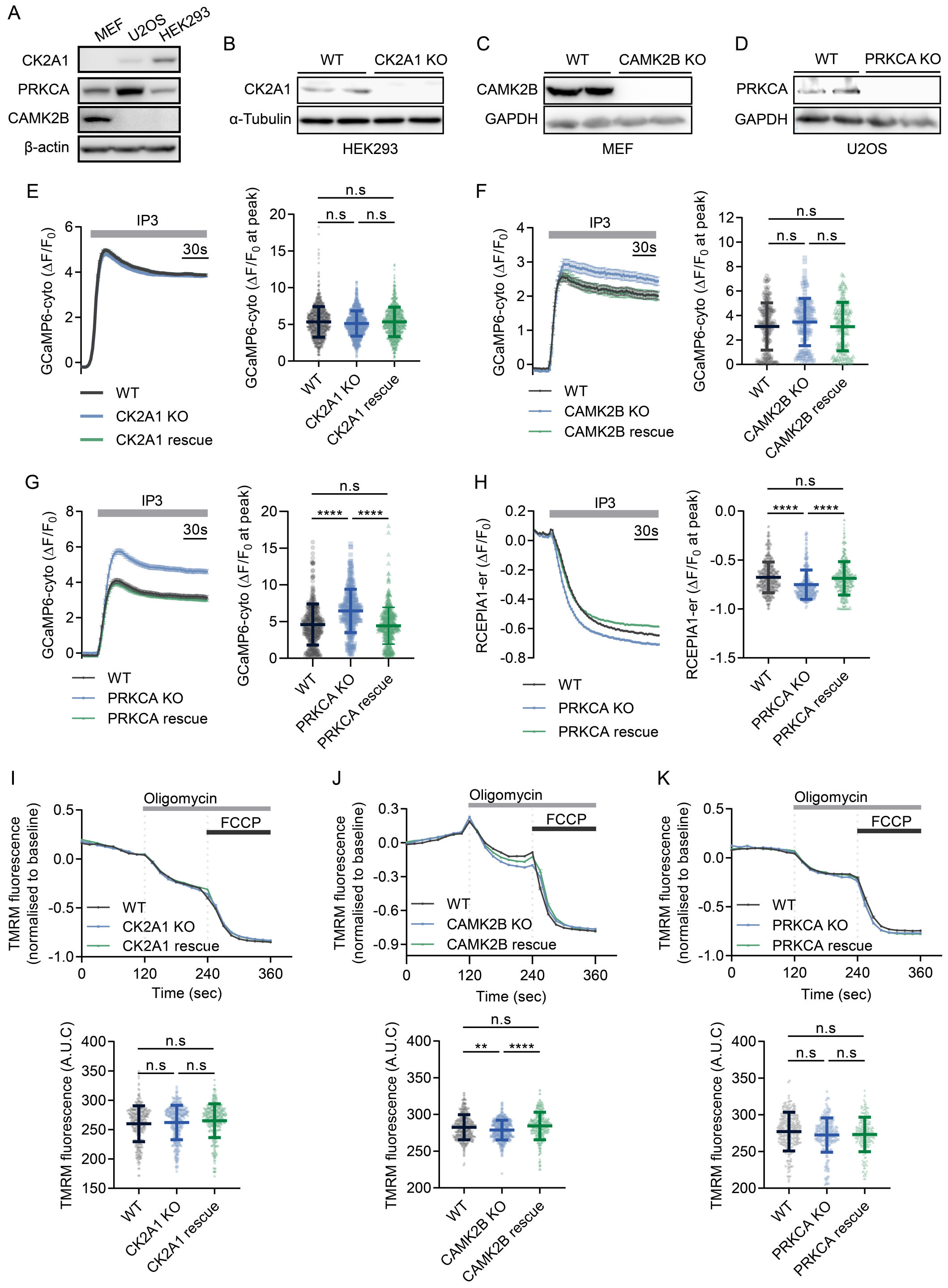

Fig S3. CK2A1, CAMK2B and PRKCA kinases modulate MAM calcium homeostasis in various manners. (A) Endogenous expression of CK2A1, CAMK2B and PRKCA kinases in MEF, U2OS and HEK293, respectively, determined by Western Blotting. (B-D) CRISPR Cas9-mediated knockout of CK2A1 (panel B), CAMK2B (panel C), and PRKCA (panel D) genes in the indicated cells and their validation by Western Blotting. (E-G) Cytosolic calcium signals in response to IP3 treatment in WT, KO and rescue cells for (E) CK2A1, (F) CAMK2B, (G) PRKCA. (H) ER calcium signals in WT, PRKCA KO and PRKCA rescue cells

after IP3 exposure. (I-K) Mitochondrial membrane potential measured by TMRM fluorescence before and after 2.5 μg/mL oligomycin and 5 μM FCCP treatment in WT, KO and rescue cells for (I) CK2A1, (J) CAMK2B and (K) PRKCA. Lower panels, quantification data of area under the curve (A.U.C) of TMRM signals. Data presented in line graphs indicate mean ± SEM. Scatter plots show mean ± SD. Each experiment was repeated at least three times. One-way ANOVA with Tukey's post hoc test for multiple comparisons was used to determine statistical significance. ns, not significant; **P<0.01; ****P<0.0001.

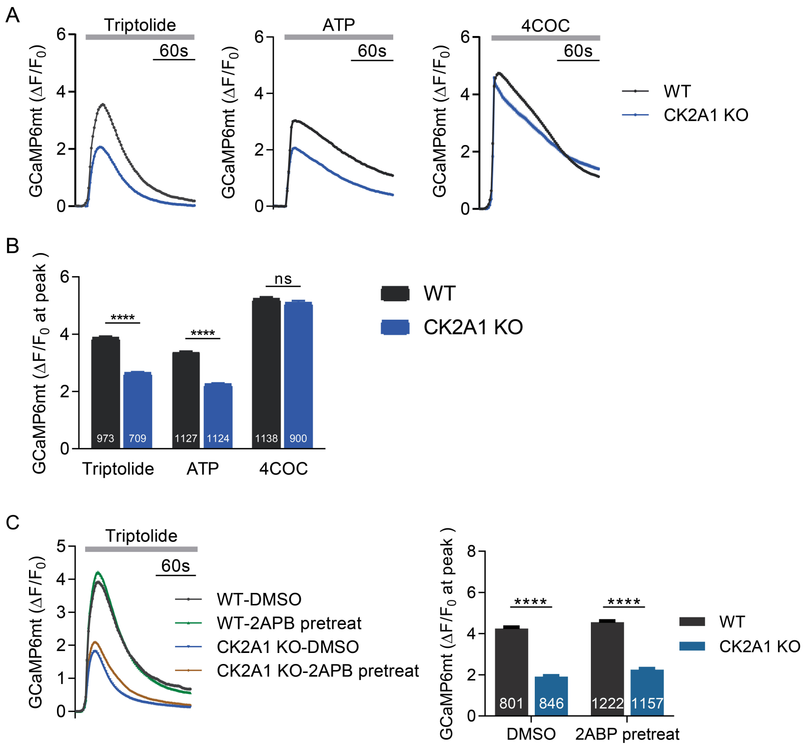

Fig S4. CK2A1 knockout diminishes mitochondrial Ca^{2+} uptake mainly through PKD2 channel. (A) Mitochondrial Ca^{2+} transients were monitored in HEK293 WT and CK2A1 KO cells before and after triptolide, ATP, and 4COC treatments. The changes in mitochondrial GCaMP6 (GCaMP6mt) (ΔF) were normalized by the baseline (F_o) before treatment. (B) Bar graph of the max peak of $\Delta F/F_o$ is presented as mean ± SEM. Total analyzed cells of each group are shown at the bottom of the bar graph. (C) After pretreatment with 2-APB or DMSO, mitochondrial Ca^{2+} signals in HEK293 WT and CK2A1 KO cells were measured upon triptolide stimulation. Right panel, bar graph shows the maximum peak of $\Delta F/ F_o$ as mean ± SEM. Total cell numbers of each group are shown at the bottom of the bars in the graph. Data in line graphs are shown as mean ± SEM. Statistical significance: ns, not significant; ****P<0.0001 by one-way ANOVA with Tukey's post hoc test for multiple comparisons.

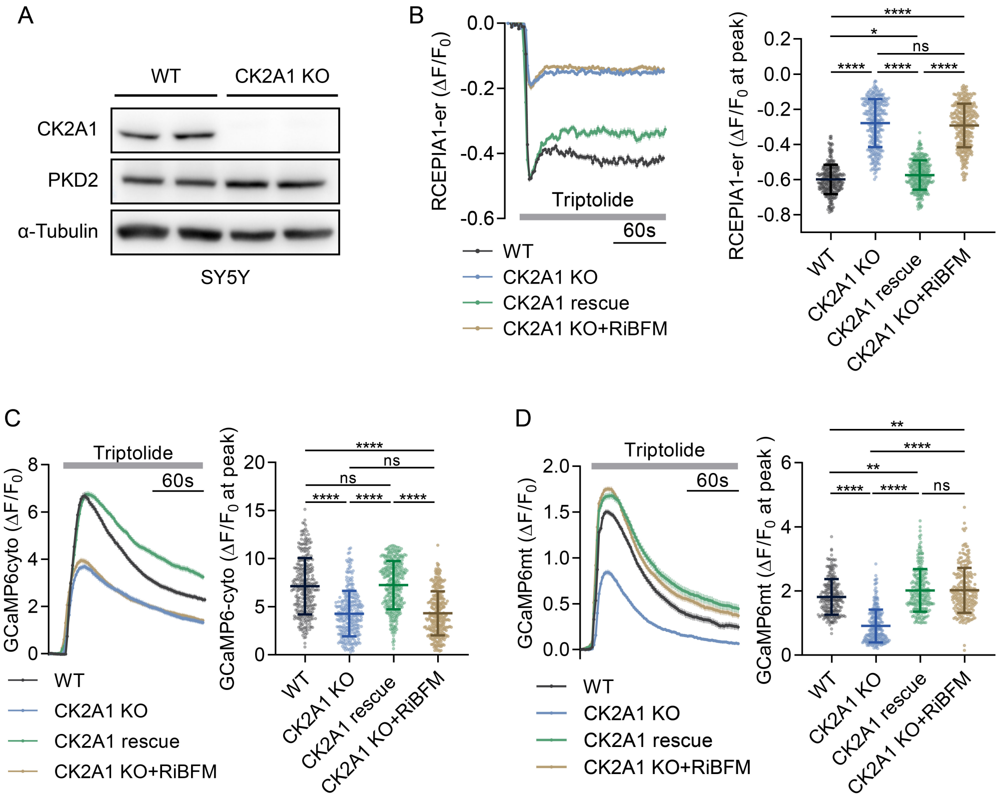

Fig S5. CK2A1 depletion impairs PKD2-mediated Ca^{2+} homeostasis at MAMs in SY5Y cells. (A) Knockout of CK2A1 in SY5Y cells was validated by western blotting. (B-D) SY5Y WT and CK2A1 KO cells were transfected with GCaMP6mt, GCaMP6cyto, or R-CEPIA1er in combination with either the vector control, CK2A1, or RiBFM plasmids to observe the changes of Ca^{2+} flux in mitochondria, cytosol, and ER, respectively. (B) Monitoring mitochondrial Ca^{2+} influx in response to triptolide stimulation. Total cells analyzed: WT = 241, CK2A1 KO = 257, CK2A1 rescue = 234, CK2A1 KO + RiBFM = 233. (C) Investigating cytosolic Ca^{2+} dynamics before and after triptolide treatment. Total cells analyzed: WT = 302, CK2A1 KO = 283, CK2A1 rescue = 328, CK2A1 KO + RiBFM = 304. (D) Tracing ER Ca^{2+} release in the indicated cells exposed to triptolide. Total cells analyzed: WT = 305, CK2A1 KO = 305, CK2A1 rescue = 298, CK2A1 KO + RiBFM = 307. Line graphs display mean ± SEM. Scatter plots show the peak amplitude of $\Delta F/F_0$ as mean ± SD. Statistical significance: ns, not significant; *P<0.05; **P<0.01; ****P<0.0001 by one-way ANOVA with Tukey's post hoc test for multiple comparisons.

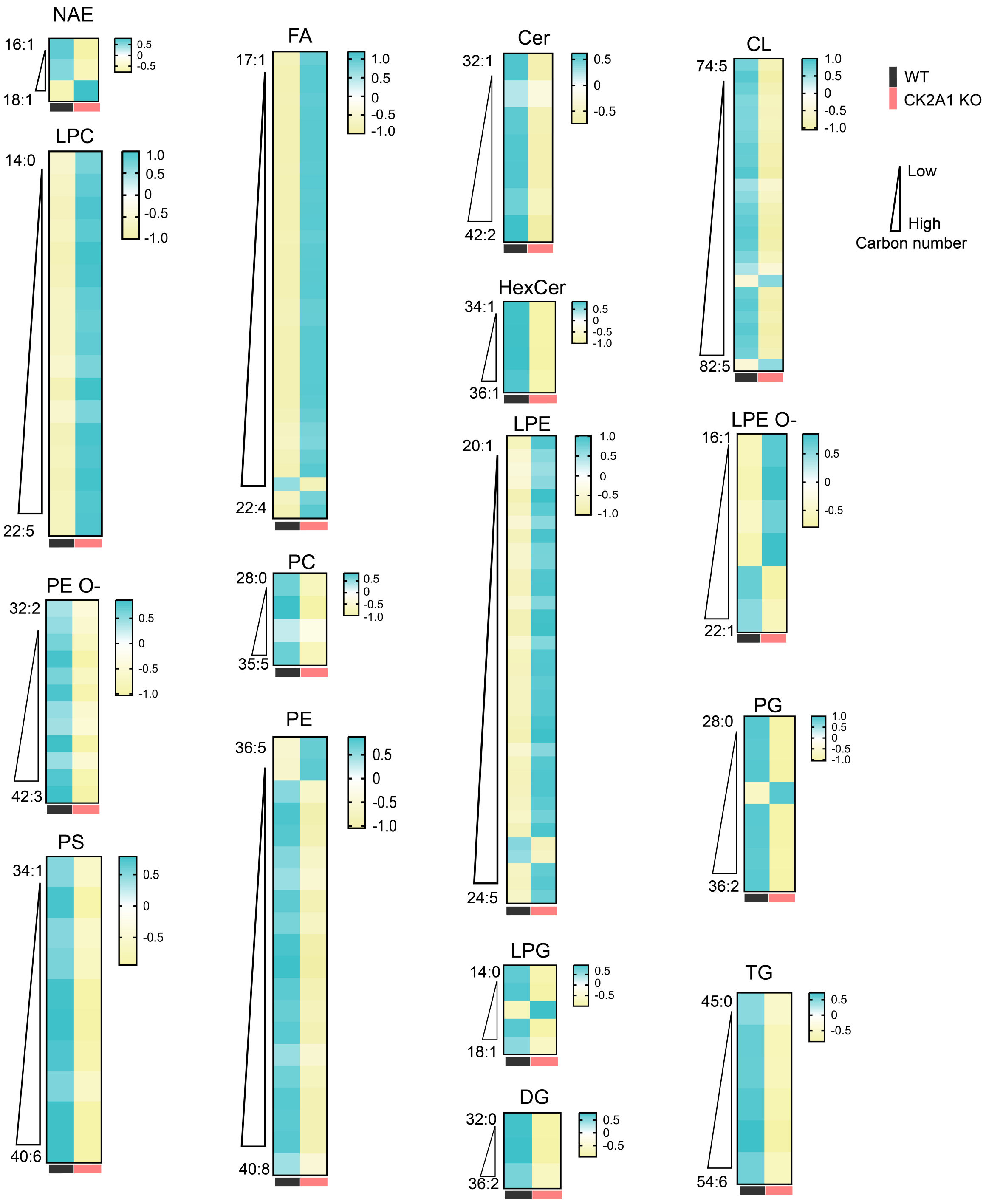

Fig S6. Expression of top lipid species significantly altered in MAM fraction between CK2A1 KO and WT cells. The mean intensity of each group is presented in the heatmap. CL: Cardiolipin, Cer: Ceramide, FA: free fatty acid, DG: Diacylglycerol, HexCer: Hexosylceramide, LPC: Lysophosphatidylcholine, LPE: Lysophosphatidylethanolamine, LPE O-: Ether-linked lysophosphatidylethanolamine, LPG: Lysophosphatidylglycerol, NAE: N-acyl ethanolamines, PC: Phosphatidylcholine, PE: Phosphatidylethanolamine, PE O-: Ether-linked phosphatidylethanolamine, PG: Phosphatidylglycerol, PS: Phosphatidylserine, TG: Triacylglycerol.

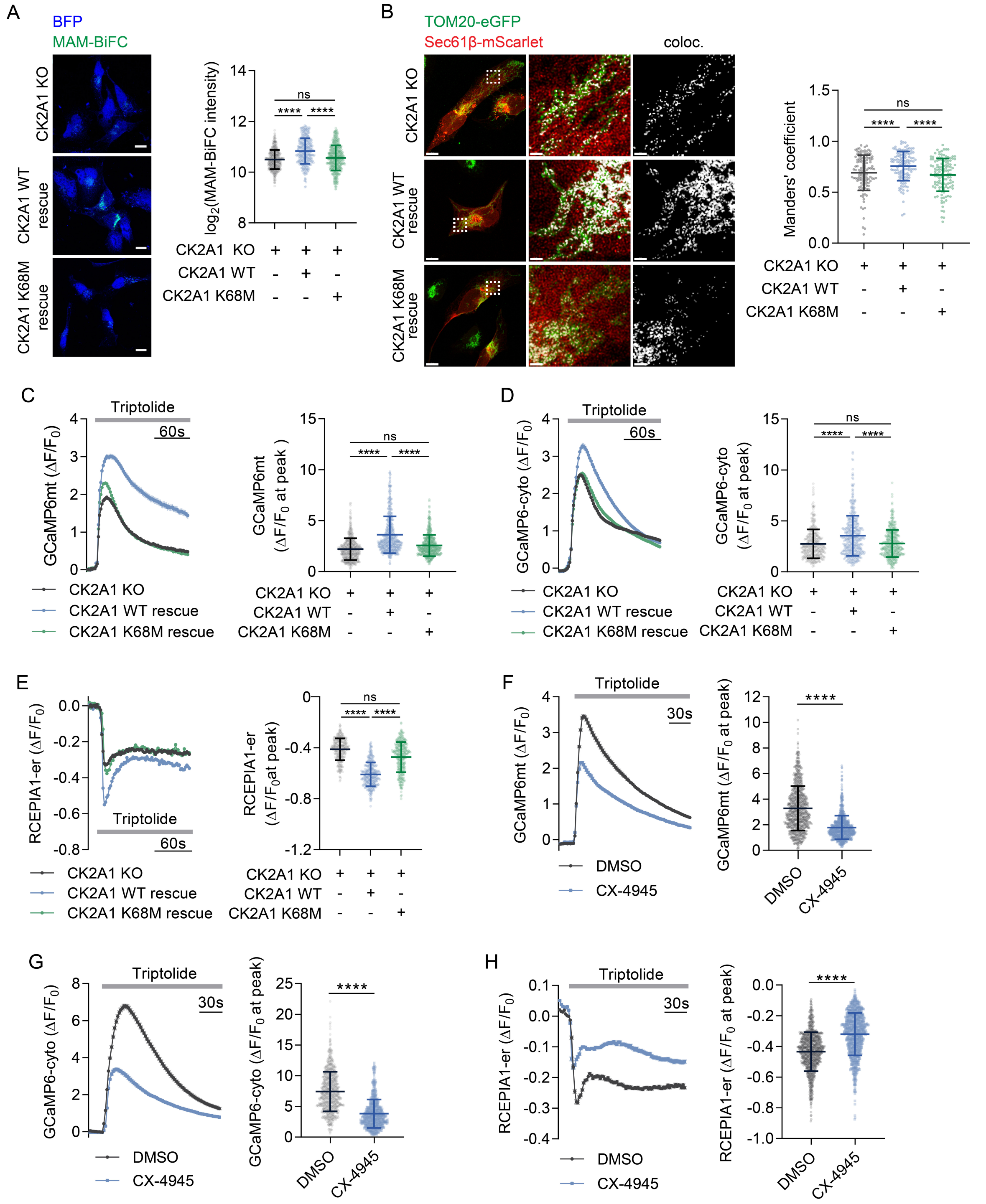

Fig S7. Catalytic activity of CK2A1 regulates the structure and Ca^{2+} homeostasis of MAMs. (A) Representative images and quantitative analysis of the MAM-BiFC intensity in the SY5Y cells lacking CK2A1 and restored with either the active or inactive (K68M) form of CK2A1. Scale bars, 25 µm. Total cells analyzed: CK2A1 KO = 474, CK2A1 WT rescue = 359, CK2A1 K68M rescue = 470. (B) Cells described in panel A were transfected with Sec61β-mScarlet plasmid and stained for TOM20 using

immunofluorescence. Scale bar = 10 µm and 1 µm for higher magnification. Quantification of Mander's coefficient is shown in scatter plot. Total cells of each group: CK2A1 KO = 124, CK2A1 WT rescue = 123, CK2A1 K68M rescue = 126. One-way ANOVA with Tukey's post hoc test for multiple comparisons was used to determine statistical significance. ns, not significant; ****P<0.0001. (C-E) The indicated cells in panel A were transfected with organelle calcium sensors (GCaMP6mt, GCaMP6cyto, and R-CEIPIA1er, respectively) and subjected to monitoring for changes in the Ca^{2+} sensor signal (ΔF) in mitochondria (C), cytosol (D), and ER (E) before and after triptolide stimulation. Total cells analyzed: GCaMP6mt (CK2A1 KO = 510; CK2A1 WT rescue = 516; CK2A1 K68M rescue = 527); GCaMP6cyto (CK2A1 KO = 416; CK2A1 WT rescue = 454; CK2A1 K68M rescue = 429); R-CEPIA1er (CK2A1 KO = 336; CK2A1 WT rescue = 348; CK2A1 K68M rescue = 337). (F-H) SY5Y cells expressing GCaMP6mt, or GCaMP6cyto, or R-CEPIA1er were pre-exposed to either DMSO or CX-4945 12 µM for 6 h to observe the changes of Ca^{2+} flux in mitochondria (panel F), cytosol (panel G), and ER (panel H), respectively, after triptolide stimulation. More than 800 cells from each group were collected and analyzed. Statistical significance: ns, not significant; *P<0.05; **P<0.01; ****P<0.0001 by two-tailed unpaired Student's t-tests. In panel C-G, line graphs display mean ± SEM and scatter plots show the peak amplitude of $\Delta F/F_o$ as mean ± SD. Each experiment was performed at least three times.

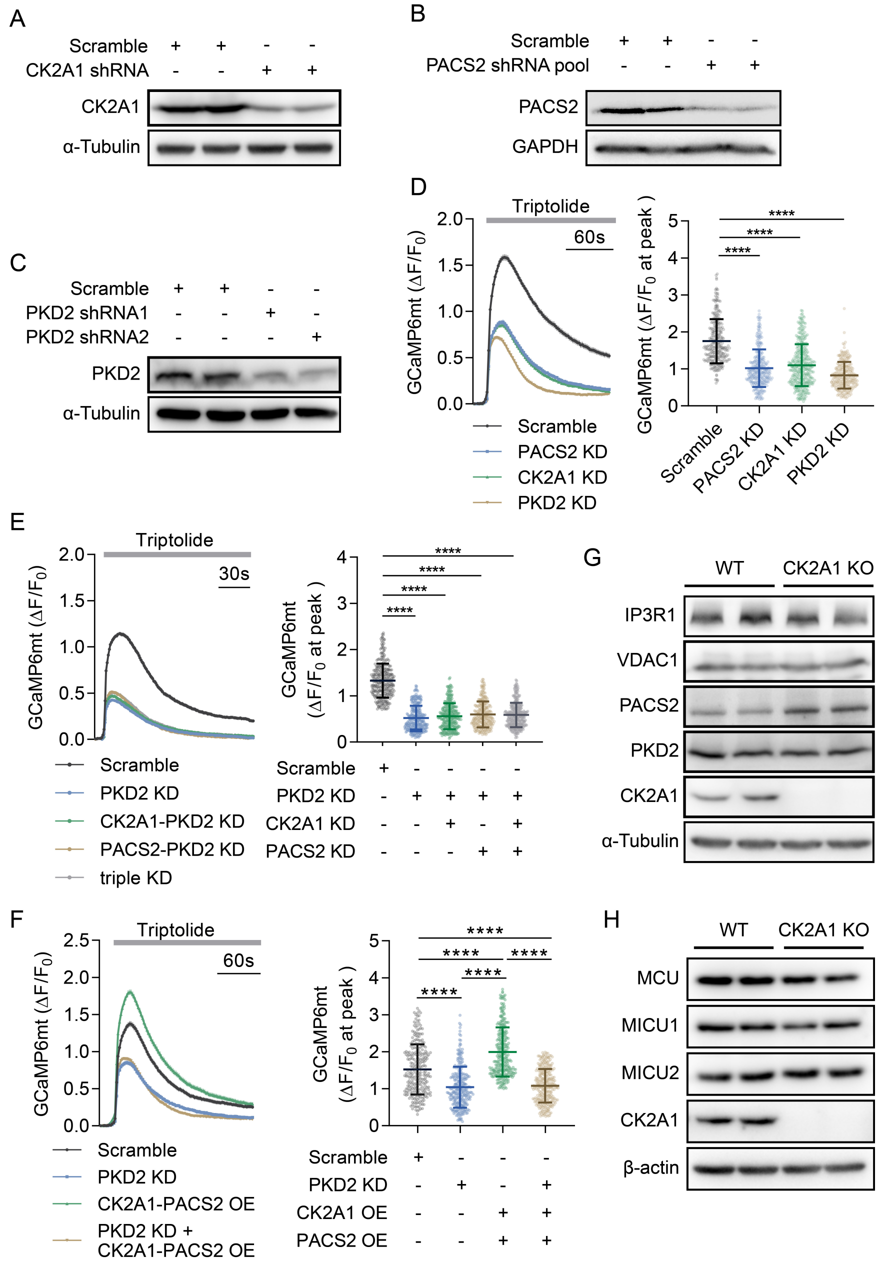

Fig S8. Regulation of MAM calcium dynamics by CK2A1 related to PACS2 and PKD2. (A-C) Western blot analysis of SY5Y cells transfected with scramble shRNA or shRNAs targeting *CK2A1* (panel A), *PACS2* (panel B), or *PKD2* (panel C), respectively. (D) Mitochondrial Ca^{2+} uptake was recorded in SY5Y cells with PKD2, CK2A1, or PACS2 knockdown before and after triptolide treatment. Total cell number of each group: scramble = 304, PACS2 knockdown (KD) = 296, CK2A1 KD = 296, PKD2 KD = 256. (E) Monitoring of the mitochondrial Ca^{2+} influx under knockdown of only PKD2 or in combination with either CK2A1, PACS2, or both. Total cell number of each group: scramble = 307, PKD2 KD = 285, PKD2-CK2A1 KD = 305, PKD2-PACS2 KD = 273, PKD2-CK2A1-PACS2 KD = 290. (F) Tracing of the mitochondrial Ca^{2+} influx in SY5Y cells transfected with PKD2 shRNAs, CK2A1, and PACS2 overexpression (OE) alone or together with PKD2 shRNAs. Total cell number of imaging: scramble = 263, PKD2 KD = 278, CK2A1-PACS2 OE = 295, CK2A1-PACS2 OE + PKD2 KD = 298. Line graphs depict

mean ± SEM. Scatter plots exhibit mean ± SD. One-way ANOVA with Tukey's post hoc test for multiple comparisons was used to determine statistical significance. ns, not significant; ****P<0.0001. (G-H) SY5Y WT and CK2A1 KO cells were collected and subjected to immunoblottings to observe the expression of the indicated proteins.

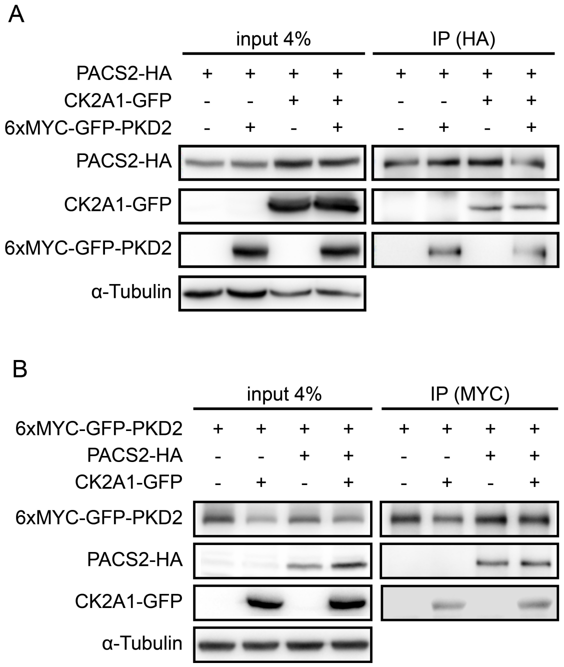

Fig S9. CK2A1 has physical interaction with PACS2 and PKD2. (A) HEK293 cells were transfected with the indicated plasmids and collected for immunoprecipitation using an anti-HA antibody, followed by western blotting. (B) After transfection with the designated plasmids, cells in panel B were harvested for immunoprecipitation using an anti-c-Myc antibody. Subsequently, western blotting was used to identify the indicated proteins.

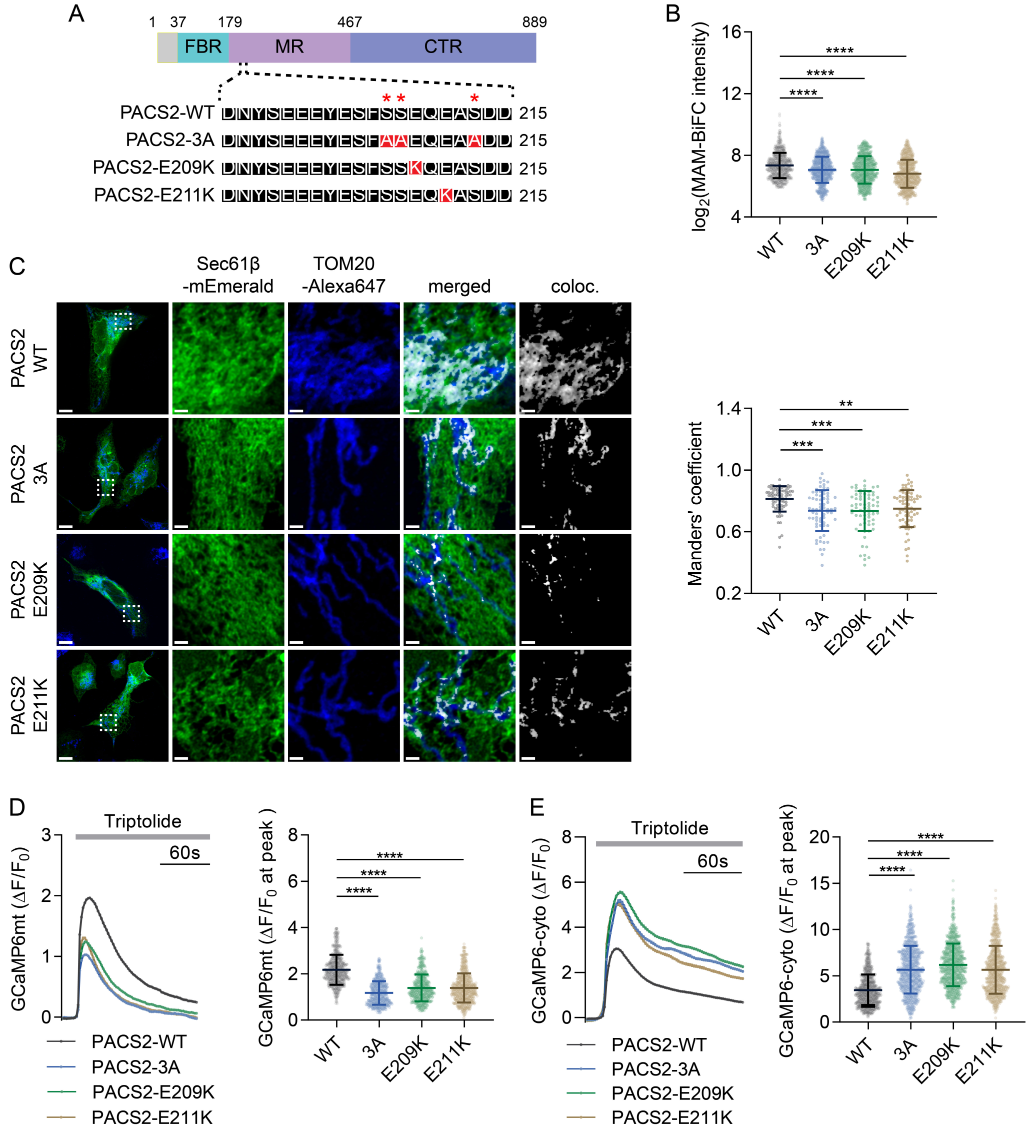

Fig S10. PACS2 phosphorylation and pathological mutations modulate MAM formation and PKD2-mediated Ca^{2+} homeostasis in SY5Y cells. (A) Diagram of PACS2 variants: PACS2 3A, E209K, and E211K. (B) MAM-BiFC intensity in cells expressing PACS2 WT or the indicated variants in panel A. Cell number of each group: PACS2 WT = 708, PACS2 3A = 775, PACS2 E209K = 724, PACS2 E211K = 711. (C) Representative images and Mander's coefficient of ER and mitochondrial markers (Sec61β and TOM20) in cells expressing PACS2 variants. Scale bars: 10 µm and 1 µm. Total cell number of each group: PACS2 WT = 74, PACS2 3A = 68, PACS2 E209K = 68, PACS2 E211K = 72. (D and E) Mitochondrial and cytosolic Ca^{2+} dynamics were recorded in the cells described in panel A upon triptolide treatment. Fluorescence changes of Ca^{2+} sensors (ΔF) were normalized by the baseline (F_o). Scatter plots show the peak of $\Delta F/F_o$.Total cells analyzed: GCaMP6mt (PACS2 WT = 759, PACS2 3A= 530, PACS2 E209K = 561, PACS2 E211K = 629), GCaMP6cyto (PACS2 WT = 1056, PACS2 3A = 900, PACS2 E209K = 911, PACS2 E211K = 826). Line graphs present mean ± SEM. Data in scatter plots are displayed as mean ± SD. Each experiment was performed independently at least three times. Statistical significance was determined by one-way ANOVA with Tukey's post hoc test for multiple comparisons. ns, not significant; **P<0.01; ***P<0.001; ****P<0.0001.

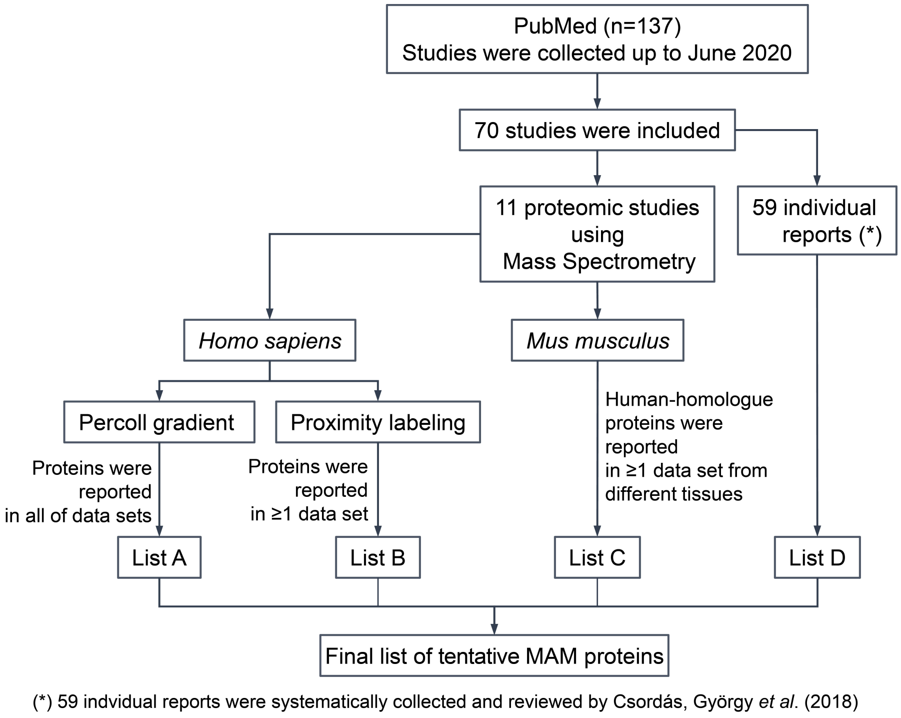

Fig S11. The workflow for extracting MAM proteome from literature sources.

Table S1. List of kinase candidates regulate MAM formation

Group	Gene ID	log_2FC	Adjusted P-value
CK2A1	1457	1.425151224	1.11E-16
DGKK	139189	1.029148097	1.11E-16
FASTKD5	60493	0.929716897	1.11E-16
GSG2	83903	0.883851459	1.11E-16
PRPF4B	8899	1.175965377	1.11E-16
PIK3C3	5289	0.989836147	1.11E-16
TEC	7006	1.257992436	2.33E-15
PRKCE	5581	0.799771391	2.89E-15
CSNK1G2	1455	0.908817087	1.69E-13
PDPK1	5170	0.865077221	2.77E-13
CCL4	6351	0.952219257	4.25E-13
ITPKB	3707	0.687307994	5.79E-13
ZAP70	7535	0.858012437	7.61E-12
PCK2	5106	0.669315504	7.98E-12
STK32C	282974	0.685264924	1.09E-11
CSNK1E	1454	0.832250228	1.20E-11
SRPK1	6732	0.792692457	2.78E-11
DCAMKL2	166614	0.723650097	3.25E-11
PLK1	5347	0.533262269	6.66E-11
PRKCA	5578	0.735565582	4.84E-10
CHKA	1119	0.729327157	5.29E-10
EIF2AK4	440275	0.499169069	8.93E-10
PDGFRB	5159	0.713818395	1.39E-09
BMP2KL	347359	0.616961416	3.02E-09
CAMK2B	816	0.697245441	4.52E-09
PRKAA2	5563	0.617458884	7.20E-09
SCYL2	55681	0.663340709	1.31E-08
FLT4	2324	0.559009176	1.39E-08
MINK1	50488	0.561557326	2.04E-08
PRKAR1A	5573	0.595095654	2.25E-08
FGFR3	2261	0.534803026	3.86E-08
EPHB6	2051	0.724275396	3.92E-08
RET	5979	0.610293656	4.28E-08
PTK2B	2185	0.624537736	5.16E-08
GTF2H1	2965	0.733223455	6.49E-08
CAMK2D	817	0.433451499	7.33E-08
MYO3B	140469	0.637042826	8.57E-08
STK24	8428	0.664034534	1.02E-07

PINK1	65018	0.709886516	2.13E-07
PDGFRA	5156	0.629663386	2.22E-07
MARK2	2011	0.530080749	2.68E-07
HCK	3055	0.569159156	4.09E-07
SNRK	54861	0.481433529	4.51E-07
PHKA2	5256	0.516116786	4.90E-07
PLXNB2	23654	0.458723489	9.68E-07
RIOK3	8780	0.499240074	1.09E-06
CSF1R	1436	0.616525626	1.32E-06
FGFR1	2260	0.542854796	1.41E-06
SLAMF6	114836	0.438067018	2.70E-06
RIPK2	8767	0.507946363	5.07E-06
EGFR	1956	0.722019071	1.05E-05
RPS6KC1	26750	0.580987346	1.15E-05
LOC340371	340371	0.550930906	1.75E-05
MAP3K8	1326	0.568480731	2.29E-05
MAP4K3	8491	0.360766172	4.70E-05
AKT3	10000	0.412735699	4.80E-05
TWF2	11344	0.437121754	5.45E-05
GRK7	131890	0.434707469	6.61E-05
FLJ23356	84197	0.441917731	7.63E-05
CK2B	1460	0.403605859	7.66E-05
CDK2	1017	0.417597563	9.22E-05
PKDCC	91461	0.430796437	9.95E-05
BMX	660	0.451672544	0.000104536
PFKFB3	5209	0.397464409	0.000134403
PIK4CA	5297	0.559835255	0.000142912
EPHA2	1969	0.482528626	0.000203676
MPP7	143098	0.415350979	0.000241975
TNK1	8711	0.477570816	0.000312182
MPP5	64398	0.589447464	0.00034625
LOC648152	545	-0.52793069	0.000350279
PHKA1	5255	0.446987969	0.000442802
PRKAB1	5564	0.415956994	0.00049642
PIP5K1B	8395	0.400526407	0.00061025
CASK	8573	0.486166019	0.000661074
MPP4	58538	0.54384984	0.000669442
PRKD1	5587	0.317387109	0.000867502
ALDH18A1	5832	0.517396532	0.001098735
HSPB8	26353	0.443539404	0.001177394

LATS2	26524	0.34655272	0.001192415
YES1	7525	0.425366683	0.001334432
IRAK2	3656	0.431972585	0.001436044
TGFBR3	7049	0.52598943	0.001690586
MAST1	22983	0.318916912	0.002005806
KHK	3795	0.48554311	0.002016307
GK	2710	0.422370878	0.002432471
MAP2K2	5605	0.353690506	0.002538949
HK3	3101	0.358191104	0.002599968
STYK1	55359	0.426929636	0.003020255
PANK4	55229	0.313686519	0.003532479
IHPK1	9807	0.399470191	0.003637128

Table S2. Database used for collection of MAM-resident proteins

**References**	**PMID**	**Tissues**	**Methods**	**Note**
Horner, Stacy M *et al*. *PloS one (2015)*	25734423	PH5CH8 and Huh7 cell lines	Percoll gradient biochemical fractionation	3005 MAM proteins of PH5CH8 cells and 1180 MAM proteins of Huh7 cells were identified in triplicate MS
Hung, Victoria *et al*. *eLife (2017)*	28441135	HEK 293T cells	APEX2 proteomic labeling scheme	68 proteins that appear in both the OMM and ERM proteomes
Cho, Il-Taeg *et al*. *J Biol chem (2017)*	28760823	HEK293T cells	mitochondrion-targeted APEX	44 proteins annotated to ER
Kwak C. *et al*. PNAS (2020)	32414919	Hek cells	Split APEX	174 MAM proteins
Kevin F. Cho *et al*. PNAS (2020)	32424107	HEK293T	Split-BioID	101 MAM proteins
Sala-Vila, Aleix *et al*. *Scientific Reports (2016)*	27272971	Mouse livers	Percoll gradient biochemical fractionation	1052 MAM-enriched proteins were identified
Ma, Jacey Hongjie, *et al*. *Scientific Reports (2017)*	28522876	Mouse brains and retina	Percoll gradient biochemical fractionation	1313 proteins, including diabetes and non-diabetes mice
Wang X *et al*. *Proteomics (2018)*	29785746	Mouse and Human Testes and mouse brains	Percoll gradient biochemical fractionation	2808 proteins from mouse testes, 2478 proteins from mouse brain, and 2155 proteins from human testes
Csordás, György *et al*. *Trends in cell biology* (2018)	29588129		Literature review	Proteins locate at MAMs, regulate MAM structure and/or functions

Table S3. Categories were used to choose kinase candidates for further validation

	CAMK2B	CK2A1	PRKCA
1. Adjusted P-value	4.52E-09	1.11E-16	4.84E-10
2. log_2(Fold change)	0.697	1.425	0.736
3. KEGG signaling pathway	Pathways of neurodegeneration - multiple diseases<br>Calcium signaling pathway<br>PI3K-Akt signaling pathway<br>Human cytomegalovirus infection<br>FoxO signaling pathway<br>Proteoglycans in cancer<br>Neurotrophin signaling pathway	Pathways of neurodegeneration - multiple diseases<br>Apoptosis<br>Mitophagy<br>Adherens junction	Pathways of neurodegeneration - multiple diseases<br>Calcium signaling pathway<br>PI3K-Akt signaling pathway<br>Salmonella infection<br>Human cytomegalovirus infection<br>Proteoglycans in cancer<br>Phosphatidylinositol signaling system<br>Autophagy<br>Apoptosis<br>Neurotrophin signaling pathway
4. Gene ontology (Biological process)	protein phosphorylation<br>protein autophosphorylation<br>regulation of vesicle-mediated transport	protein phosphorylation	protein phosphorylation<br>membrane organization<br>protein autophosphorylation<br>regulation of cellular response to stress
5. Protein-protein interaction (with published MAM proteins)	CAMK2D, PIK3C3, CPT1A, LIN7C, ITPR1, CDC42, FLNC, PTK2B, CAV1, DLG, RAC1, CALM1, ITPR3, FLNB, LIN7A	APOE, C1QBP, CALM1, CAV1, CDC5L, CHD4, CHEK1, CHKA, CSNK1E, CSNK2B, DCLK2, EGFR, HMGA1, HMOX2, HSPB8, IGF2R, JUND, MTOR, MYH10, MYH9, NAP1L4, NME3, PACS2, PKD2, PRKAR1A, PRKDC, PSEN2, PTPN1, SEC63, SPTBN1	ARF5, GNA11, ITPKB, TJP1, GNAQ, PA2G4, HSPB8, ITGB1, PRKCE, ITPR1, HMGA1, GNAI2, C1QBP, PRKDC, CDC42, YES1, GSK3B, FLNC, ACTG1, PTK2B, PRKD1, AKAP1, RHOG, CD82, HMGB1, PDPK1, ANXA6, RET, RAC1, FAS, RICTOR, PRKAR1A, MTOR, LMNA, LMNB1, GNAI3, FLNA, ITPR3, TEC, ITPR2, MLST8, CHEK1, RHOA, ADCY5, PIK3C3, DBI, MAP2K2, ICAM1, MAPKAP1, EGFR

Table S4. Significantly altered lipids of MAM isolated from CK2A1 KO samples as compared to WT

Lipid name	FDR	log2(Fold Change)
CAR 14:0	7.68E-06	-0.97715
Cer 32:1;O2|Cer 18:1;O2/14:0	0.002705	-0.41671
Cer 33:1;O2|Cer 17:1;O2/16:0	0.005345	-0.10904
Cer 33:1;O2|Cer 18:1;O2/15:0	0.006408	-0.34071
Cer 36:2;O2|Cer 18:2;O2/18:0	0.003425	-0.45746
Cer 38:2;O2|Cer 18:2;O2/20:0	0.002883	-0.55582
Cer 42:0;O2|Cer 18:0;O2/24:0	0.02157	-0.38531
Cer 42:2;O2|Cer 18:2;O2/24:0	0.000642	-0.69191
CL 66:2|CL 16:0_16:0_16:0_18:2	1.02E-05	-0.32114
CL 66:3|CL 18:0_16:1_16:1_16:1	1.69E-13	-1.6157
CL 66:5|CL 16:1_16:1_16:1_18:2	6.26E-07	-0.83363
CL 68:1|CL 14:0_14:0_24:0_16:1	0.000139	-0.28554
CL 68:6|CL 16:1_16:1_16:1_20:3	6.59E-05	-0.53857
CL 69:2|CL 16:0_17:0_18:1_18:1	0.000127	-0.43418
CL 69:3|CL 16:0_16:0_17:0_20:3	2.23E-05	-0.54201
CL 69:4|CL 17:1_17:1_17:1_18:1	1.61E-08	-0.91793
CL 70:1|CL 14:0_14:0_26:0_16:1	5.24E-08	-0.68719
CL 70:3|CL 16:0_18:1_18:1_18:1	1.21E-15	-1.2033
CL 70:7|CL 16:1_18:2_18:2_18:2	0.009845	-0.28914
CL 71:3|CL 16:0_17:0_17:0_21:3	0.000732	-0.40886
CL 71:5|CL 18:1_18:1_18:1_17:2	1.31E-06	-0.78062
CL 72:1|CL 16:0_16:0_24:0_16:1	1.88E-09	-0.57304
CL 72:3|CL 18:0_18:1_18:1_18:1	1.12E-12	-0.93549
CL 74:5|CL 16:0_18:0_18:0_22:5	3.34E-09	-0.66278
CL 74:9|CL 18:2_18:2_18:2_20:3	9.38E-05	-0.43631
CL 76:1|CL 16:0_16:0_26:0_18:1	0.028179	-0.2068
CL 76:1|CL 17:0_17:0_24:0_18:1	0.006032	0.34062
CL 76:3|CL 18:0_18:0_18:0_22:3	1.01E-07	-0.73897
CL 76:3|CL 24:0_17:1_17:1_18:1	9.45E-11	-0.67763
CL 78:1|CL 16:0_16:0_28:0_18:1	3.64E-07	-0.62853
CL 78:3|CL 24:0_18:1_18:1_18:1	9.22E-12	-0.58364
CL 78:3|CL 26:0_16:1_18:1_18:1	1.36E-07	-0.66273
CL 78:5|CL 26:0_16:1_16:1_20:3	4.27E-06	-0.47327

CL 82:5|CL 26:0_18:1_18:1_20:3	0.009936	0.15486
CoQ10	0.001506	-0.82251
DG 32:0|DG 16:0_16:0	2.14E-06	-1.4908
DG 32:1|DG 14:0_18:1	7.68E-07	-1.2911
DG 36:2|DG 16:1_20:1	0.003849	-0.90212
DG O-38:6|DG O-18:2_20:4	0.016129	0.87992
DG O-38:7|DG O-16:1_22:6	0.001273	0.95798
FA 14:0	8.66E-08	1.3316
FA 15:0	3.09E-12	1.9122
FA 16:0	5.00E-12	1.0556
FA 16:1	1.88E-09	1.8006
FA 16:2	1.16E-10	2.0903
FA 17:0	5.82E-12	1.8246
FA 17:1	4.09E-11	1.6372
FA 17:2	5.17E-11	2.1901
FA 18:0	3.08E-10	0.88337
FA 18:1	5.00E-12	1.6844
FA 18:1 (2)	2.06E-10	1.2829
FA 18:2	1.53E-11	2.0696
FA 18:2 (2)	2.65E-12	2.6265
FA 18:2;O	1.52E-08	0.96887
FA 18:3	2.29E-10	1.8206
FA 19:0	9.53E-11	1.3856
FA 19:2	1.53E-11	1.8469
FA 19:3	5.82E-12	2.6493
FA 20:0	3.57E-08	0.44396
FA 20:1	9.52E-08	0.98705
FA 20:2	2.24E-11	1.993
FA 20:3	2.76E-12	2.5676
FA 20:3 (2)	4.12E-12	2.5841
FA 20:4	5.82E-12	2.4505
FA 20:4;O	7.94E-13	1.8286
FA 20:5	9.45E-11	2.1402
FA 20:5;O	4.96E-08	2.0702
FA 22:0	1.67E-05	0.3274
FA 22:1	7.32E-05	0.42924

FA 22:2	4.26E-08	1.0042
FA 22:6	1.43E-12	1.895
FA 24:1	0.000105	-0.32718
FA 24:3	1.21E-05	0.64447
FA 24:4	1.53E-11	1.5019
HexCer 34:1;O2|HexCer 18:1;O2/16:0	1.35E-08	-0.87836
HexCer 34:7;2O|HexCer 16:2;O2/18:5	7.20E-10	-1.0915
HexCer 35:7;2O|HexCer 17:2;O2/18:5	3.08E-10	-0.91073
HexCer 36:1;O2|HexCer 18:1;O2/18:0	2.52E-06	-0.66087
LPC 14:0	0.001465	0.52815
LPC 14:0 (2)	7.36E-05	0.61467
LPC 15:0	1.76E-06	0.92929
LPC 16:0	3.66E-05	0.73378
LPC 17:0	1.17E-07	1.2818
LPC 17:0 (2)	7.68E-07	0.98362
LPC 18:0	6.54E-05	0.44381
LPC 18:1	0.000235	0.85584
LPC 18:2	6.65E-05	1.0501
LPC 18:3	0.002178	0.71797
LPC 19:1	2.06E-08	1.202
LPC 20:0	0.002331	0.27486
LPC 20:1	4.27E-07	0.9769
LPC 20:4	2.85E-06	1.0098
LPC 22:4/0:0	1.53E-07	1.7685
LPC 22:5	5.50E-06	0.89383
LPC 22:5 (2)	2.85E-06	1.2875
LPC O-18:1	5.34E-05	0.69043
LPC P-18:0	1.66E-05	0.85712
LPE 16:0	2.14E-06	0.72774
LPE 16:0 (2)	0.006758	0.48359
LPE 16:1	0.029709	0.26849
LPE 16:1 (2)	0.009689	0.40986
LPE 17:0	1.16E-09	1.2153
LPE 17:1	1.09E-05	0.76892
LPE 17:1 (2)	0.006798	0.46867
LPE 18:0	4.27E-07	0.92174

LPE 18:1	0.001301	0.62821
LPE 18:1 (2)	0.001377	0.47463
LPE 18:2	1.64E-06	0.77061
LPE 18:2 (2)	7.68E-07	0.97747
LPE 18:3	0.003621	0.49075
LPE 19:1	2.85E-09	0.86852
LPE 20:0	3.64E-09	1.2226
LPE 20:1	0.003887	0.421
LPE 20:2	2.24E-06	0.72844
LPE 20:3	1.87E-06	0.73269
LPE 20:3 (2)	2.63E-05	0.67619
LPE 20:4	7.68E-06	0.91719
LPE 20:4 (2)	7.68E-06	0.69285
LPE 22:0	3.61E-08	0.68275
LPE 22:1	9.68E-11	1.1317
LPE 22:2	0.005769	0.41614
LPE 22:3	5.50E-06	0.8379
LPE 22:4	1.31E-06	0.86586
LPE 22:4 (2)	1.50E-06	1.1658
LPE 22:5	2.41E-05	0.63484
LPE 22:5 (2)	0.000773	0.75493
LPE 22:6	6.22E-07	0.84388
LPE 24:0	0.00026	-0.52285
LPE 24:1	0.002705	-0.22286
LPE 24:4	3.77E-05	0.74569
LPE 24:5	7.68E-06	0.76743
LPE 24:5 (2)	0.000356	0.71777
LPE O-16:1	0.001009	0.83619
LPE O-17:1	7.62E-05	0.96265
LPE O-18:1	0.004393	0.43472
LPE O-18:2	3.43E-05	1.0859
LPE O-20:1	0.000183	-0.21766
LPE O-22:1	0.007967	-0.16685
LPE P-18:0	0.001301	0.7131
LPG 14:0	4.77E-07	-2.1436
LPG 17:1	1.06E-09	-2.1465

LPG 18:0	4.24E-07	1.038
LPG 18:1	1.51E-08	-1.9488
LPG 18:1 (2)	0.00265	-0.73378
NAE 16:1	0.000323	-0.38554
NAE 18:0	0.008349	-0.21206
NAE 18:1	0.000261	0.86116
PC 17:0/17:0	0.015595	-0.0887
PC 28:0	0.000549	-0.64995
PC 33:4	3.64E-09	-0.87946
PC 35:5|PC 15:1_20:4	0.000323	-0.62202
PC O-16:0/18:1	4.40E-08	-0.64384
PC O-36:2	0.024544	-0.28383
PC O-40:6|PC O-22:6_18:0	0.044188	0.12952
PC P-18:1/20:4	1.42E-06	-0.38374
PC-O 16:0/18:1	2.94E-06	-0.49351
PE 17:0/17:0	0.001537	0.56782
PE 32:0	0.000937	0.61889
PE 32:1	0.004331	-0.4393
PE 32:1|PE 14:0_18:1	9.66E-09	-0.45224
PE 32:2|PE 16:1_16:1	4.85E-07	-0.5294
PE 34:1	0.002993	-0.40371
PE 35:1	0.020624	-0.40919
PE 35:2	7.68E-06	-0.86371
PE 36:1	0.000872	-0.6677
PE 36:2	1.12E-08	-1.0089
PE 36:2|PE 18:1_18:1	6.12E-14	-1.1153
PE 36:3	7.49E-06	-0.81159
PE 36:5	8.59E-05	-0.86737
PE 38:6	3.62E-06	-1.0604
PE 38:6 (2)	0.018204	-0.3256
PE 38:7	0.000217	-0.8121
PE 40:6	2.00E-06	-0.45163
PE 40:6 (2)	7.60E-06	-0.87234
PE 40:7	2.20E-06	-0.67339
PE 40:8	0.032262	-0.47705
PE O-33:2|PE O-16:1_17:1	0.049831	-0.0892

PE O-35:2|PE O-17:1_18:1	0.015058	-0.11965
PE O-36:6	0.001032	-0.29205
PE O-36:6|PE O-18:5_18:1	3.74E-09	-0.99496
PE O-36:7|PE O-18:5_18:2	0.00066	-0.44493
PE O-37:2|PE O-18:1_19:1	5.37E-08	-0.44604
PE O-37:3	0.017626	-0.30002
PE O-37:4|PE O-16:1_21:3	0.034071	-0.16722
PE O-38:3|PE O-18:2_20:1	5.72E-11	-0.62769
PE O-38:6	0.02467	-0.27575
PE O-40:6|PE O-20:2_20:4	3.64E-07	-0.2876
PE O-42:3|PE O-24:2_18:1	8.32E-12	-0.82375
PE P-18:1/18:1	2.72E-09	-0.4689
PE P-34:1|PE P-18:1_16:0	0.025654	-0.32612
PEtOH 32:2|PEtOH 16:1_16:1	3.08E-10	-2.448
PEtOH 33:1|PEtOH 17:0_16:1	0.013038	-2.2487
PG 17:0/17:0	4.66E-13	-1.5061
PG 28:0|PG 14:0_14:0	1.68E-13	-2.1933
PG 32:0	6.88E-10	-1.4079
PG 32:0|PG 16:0_16:0	4.00E-16	-1.2697
PG 32:2|PG 16:1_16:1	7.46E-06	1.1
PG 34:2|PG 16:1_18:1	5.99E-12	-1.758
PG 34:3|PG 16:1_18:2	4.80E-10	-1.2187
PG 36:2	2.64E-11	-1.4604
PI 17:1/14:1	6.75E-10	-1.115
PI 36:5	3.66E-10	-1.2093
PMeOH 42:2|PMeOH 21:1_21:1	0.044224	-0.48852
PS 34:1	0.011853	-0.24375
PS 36:1	5.87E-06	-0.55623
PS 36:2	0.011862	-0.33023
PS 38:2	0.004377	-0.30518
PS 38:6	2.52E-06	-0.59993
PS 40:1	7.68E-07	-0.69601
PS 40:2	1.72E-05	-0.55903
PS 40:4	0.007081	-0.69085
PS 40:5	1.17E-06	-0.70437
PS 40:6	1.18E-06	-0.94711

SM 41:2;O2	4.25E-08	-1.2417
Sphinganine (d18:0)	0.011542	0.21693
Sphingosine (d18:1)	0.03759	0.18247
TG 45:0	0.030966	-0.2471
TG 46:0	0.002735	-0.36588
TG 46:2	0.003279	-0.3382
TG 50:2	0.000538	-0.57092
TG 51:1	2.18E-05	-0.84776
TG 54:6	0.006297	-0.27636

References

1. J. Lu *et al.*, Polycystin-2 Plays an Essential Role in Glucose Starvation-Induced Autophagy in Human Embryonic Stem Cell-Derived Cardiomyocytes. *Stem Cells* **36**, 501-513 (2018).
2. S. J. Park *et al.*, DISC1 Modulates Neuronal Stress Responses by Gate-Keeping ER-Mitochondria Ca(2+) Transfer through the MAM. *Cell Rep* **21**, 2748-2759 (2017).
3. D. Mashiko *et al.*, Generation of mutant mice by pronuclear injection of circular plasmid expressing Cas9 and single guided RNA. *Sci Rep* **3**, 3355 (2013).
4. E. Herberich, J. Sikorski, T. Hothorn, A robust procedure for comparing multiple means under heteroscedasticity in unbalanced designs. *PLoS One* **5**, e9788 (2010).
5. I. Annunziata, A. Patterson, A. d'Azzo, Mitochondria-associated ER membranes (MAMs) and glycosphingolipid enriched microdomains (GEMs): isolation from mouse brain. *J Vis Exp*, e50215 (2013).
6. M. R. Wieckowski, C. Giorgi, M. Lebiedzinska, J. Duszynski, P. Pinton, Isolation of mitochondria-associated membranes and mitochondria from animal tissues and cells. *Nat Protoc* **4**, 1582-1590 (2009).
7. T. Simmen *et al.*, PACS-2 controls endoplasmic reticulum-mitochondria communication and Bid-mediated apoptosis. *EMBO J* **24**, 717-729 (2005).
8. A. Provost *et al.*, Innovative particle standards and long-lived imaging for 2D and 3D dSTORM. *Sci Rep* **9**, 17967 (2019).
9. K. Jaqaman *et al.*, Robust single-particle tracking in live-cell time-lapse sequences. *Nat Methods* **5**, 695-702 (2008).
10. I. Belevich, M. Joensuu, D. Kumar, H. Vihinen, E. Jokitalo, Microscopy Image Browser: A Platform for Segmentation and Analysis of Multidimensional Datasets. *PLoS Biol* **14**, e1002340 (2016).
11. P. Cosson, A. Marchetti, M. Ravazzola, L. Orci, Mitofusin-2 independent juxtaposition of endoplasmic reticulum and mitochondria: an ultrastructural study. PLoS One 7, e46293 (2012).
12. E. Tubbs, J. Rieusset, Study of Endoplasmic Reticulum and Mitochondria Interactions by In Situ Proximity Ligation Assay in Fixed Cells. J Vis Exp 10.3791/54899 (2016).
13. E. Tubbs et al., Mitochondria-associated endoplasmic reticulum membrane (MAM) integrity is required for insulin signaling and is implicated in hepatic insulin resistance. Diabetes 63, 3279-3294 (2014).
14. N. H. Anh *et al.*, Caenorhabditis elegans deep lipidome profiling by using integrative mass spectrometry acquisitions reveals significantly altered lipid networks. *J Pharm Anal* **12**, 743-754 (2022).
15. D. K. Barupal *et al.*, Generation and quality control of lipidomics data for the Alzheimer's disease neuroimaging initiative cohort. *Sci Data* **5**, 180263 (2018).
16. H. Tsugawa *et al.*, A lipidome atlas in MS-DIAL 4. *Nat Biotechnol* **38**, 1159-1163 (2020).
17. G. Liebisch *et al.*, Update on LIPID MAPS classification, nomenclature, and shorthand notation for MS-derived lipid structures. *J Lipid Res* **61**, 1539-1555 (2020).
18. Z. Pang *et al.*, MetaboAnalyst 5.0: narrowing the gap between raw spectra and functional insights. *Nucleic Acids Res* **49**, W388-W396 (2021).
19. W. J. Lin *et al.*, LipidSig: a web-based tool for lipidomic data analysis. *Nucleic Acids Res* **49**, W336-W345 (2021).
